# Microarray analysis of bone marrow lesions in osteoarthritis demonstrates upregulation of genes implicated in osteochondral turnover, neurogenesis and inflammation

**DOI:** 10.1136/annrheumdis-2017-211396

**Published:** 2017-07-13

**Authors:** Anasuya Kuttapitiya, Lena Assi, Ken Laing, Caroline Hing, Philip Mitchell, Guy Whitley, Abiola Harrison, Franklyn A Howe, Vivian Ejindu, Christine Heron, Nidhi Sofat

**Affiliations:** 1 Institute for Infection & Immunity, St George’s, University of London, London, UK; 2 St George’s University Hospitals NHS Foundation Trust, London, UK; 3 Institute for Molecular and Clinical Sciences, St George’s, University of London, London, UK

**Keywords:** Knee Osteoarthritis, Magnetic Resonance Imaging, Inflammation, Chondrocytes, Chemokines

## Abstract

**Objective:**

Bone marrow lesions (BMLs) are well described in osteoarthritis (OA) using MRI and are associated with pain, but little is known about their pathological characteristics and gene expression. We evaluated BMLs using novel tissue analysis tools to gain a deeper understanding of their cellular and molecular expression.

**Methods:**

We recruited 98 participants, 72 with advanced OA requiring total knee replacement (TKR), 12 with mild OA and 14 non-OA controls. Participants were assessed for pain (using Western Ontario and McMaster Universities Osteoarthritis Index (WOMAC)) and with a knee MRI (using MOAKS). Tissue was then harvested at TKR for BML analysis using histology and tissue microarray.

**Results:**

The mean (SD) WOMAC pain scores were significantly increased in advanced OA 59.4 (21.3) and mild OA 30.9 (20.3) compared with controls 0.5 (1.28) (p<0.0001). MOAKS showed all TKR tissue analysed had BMLs, and within these lesions, bone marrow volume was starkly reduced being replaced by dense fibrous connective tissue, new blood vessels, hyaline cartilage and fibrocartilage. Microarray comparing OA BML and normal bone found a significant difference in expression of 218 genes (p<0.05). The most upregulated genes included stathmin 2, thrombospondin 4, matrix metalloproteinase 13 and Wnt/Notch/catenin/chemokine signalling molecules that are known to constitute neuronal, osteogenic and chondrogenic pathways.

**Conclusion:**

Our study is the first to employ detailed histological analysis and microarray techniques to investigate knee OA BMLs. BMLs demonstrated areas of high metabolic activity expressing pain sensitisation, neuronal, extracellular matrix and proinflammatory signalling genes that may explain their strong association with pain.

## Introduction

Osteoarthritis (OA) is the most common form of arthritis worldwide affecting more than 27 million adults in the USA alone^1^ and is a major cause of pain and functional disability. OA prevalence is set to rise globally with ageing populations accompanied by the rising epidemic of obesity.^2^ OA most commonly affects large weight-bearing joints, affecting the knees in up to 37% of adults over 60.^1^ Pain is a major symptom for people with OA, with 16.7% of US adults aged 45 years and above reporting pain as a predominant problem.^1^

Pain in OA is thought to arise from several structures within the arthritic joint, including the synovium (from which prostaglandins, leukotrienes and inflammatory mediators are released), joint effusions, joint capsule involvement, tendon and muscle weakness that all contribute to pain and reduced function.^3^ Synovitis is often observed by MRI in OA and strongly correlates with pain.^4^ Cartilage degradation is one of the hallmarks of OA disease^5^ and exposes the structures from which pain is most likely arising as cartilage is an avascular, aneural structure composed largely of extracellular matrix (ECM) embedded sparsely with chondrocytes. Recent interest has grown in the importance of bone marrow lesions (BMLs) in relation to pain in OA. Epidemiological studies have shown a strong correlation between BMLs observed by MRI and OA-related knee pain in several large cohorts,^6 7^ with an OR of 3.2 for the association of BMLs with pain. The data outlined above demonstrate the multifactorial nature of OA and how pain mechanisms are supported by the biopsychosocial model of pain.

Recently, BMLs have been shown to be a very early biomarker of joint damage in OA^6 7^ with descriptions of their histology and histomorphometry. However, no previous transcriptomic studies of BMLs in OA are described. In the current study, we describe novel findings demonstrating BMLs have features of angiogenesis, fibrosis, new cartilage formation and increased bone turnover with disruption of the physiological osteochondral interface. Whole transcriptomic analysis of BML regions found upregulated expression of genes involved in neurogenesis, pain sensitisation, chemokine and cytokine signalling as well as cartilage remodelling pathways.

## Materials and methods

All study procedures were carried out after ethical approval was granted (Health Research Authority approval number 12/LO/1970 and clinical trials.gov identifier NCT02603939). Participants attending the South London Elective Orthopaedic Centre were recruited at assessment for total knee replacement (TKR), comprising the ‘advanced OA group’. For the ‘mild OA’ group, participants were recruited from rheumatology clinics at St George’s University Hospitals NHS Foundation Trust. For bone tissue controls, participants undergoing surgery following trauma, amputation or trochleoplasty were recruited (approval number 09/H0806/45) with no clinical or radiographic arthritis. Blood and urine samples were also obtained with full consent for biomarker studies.

### Study criteria

Eligibility for participation included age of 35–90 years, presenting with pain and fulfilling ACR criteria for the diagnosis of knee OA.^8^ Participants continued to experience pain despite treatment for OA.^9^ All participants underwent baseline knee radiography to confirm knee OA with a Kellgren-Lawrence grade of greater than 2 in the affected tibio-femoral knee joint.^10^

### Clinical data collection

All scores were collected for participants with advanced OA and mild OA. For controls, Western Ontario and McMaster Universities Osteoarthritis Index (WOMAC) was not collected as participants underwent different surgeries. The primary pain score was the WOMAC with subscales for pain, stiffness and function.^11^ Participants were asked to score based on symptoms in the last 48 hours. Data were also collected for body mass index (BMI), Visual Analogue Scale pain rating 0–10^12^ and the Hospital Anxiety and Depression Scale.^13^

### Molecular methods

Total RNA was isolated from approximately 200 mg of bone tissue. Amplified labelled cRNA samples (600 ng) were hybridised to Agilent whole human genome 60 k microarray chips. Array signal intensities were analysed by the Agilent Gene-Spring GX software. Significant differentially expressed entities between bone samples from healthy controls and OA participants were selected using a union of a Student’s/moderated t-test corrected for multiple comparisons with the Bonferroni correction (p<0.05). Further methodical details are provided in the online supplementary methods. 14–17

10.1136/annrheumdis-2017-211396.supp1 Supplementary file 1

## Statistical analysis

Data were anonymised for all analyses independently by the research team who were not involved in diagnosing or treating the study participants. To detect significant differences between groups at p<0.05, recruitment of at least 80 subjects was required, and we achieved n=98 participants. GraphPad Prism V.7 was used for all analyses, and significance was set at p<0.05 for all analyses. For microarray statistical analysis, refer to online supplementary methods.

## Results

Demographic data showed that our participants were representative of a knee OA population. Knee OA participants who underwent TKR had a high BMI and high pain scores measured by WOMAC (table 1). The mean (SD) WOMAC pain scores were significantly increased in advanced OA 59.4 (21.3) and mild OA 30.9 (20.3) compared with controls 0.5 (1.28) (p<0.0001), showing the advanced OA group had significantly more severe pain and functional impairment.

**Table 1:** Demographics showing characteristics of study population key. Data presented as means and SD

		Advanced OA	Mild OA	Tissue control
Number*		72	12	10
Age range Mean (SD)		51–88 69.1 (7.7)	49–79 62.2 (8.5)	21–88 56.2 (27.7)
Gender Female N (%)		55 (76.4)	9 (75)	9 (90)
Body mass index Mean (SD)		32.5 (5.7)	28.8 (3.9)	N/A
WOMAC pain Mean (SD)		59.4 (21.3)	30.9 (20.3)	N/A
WOMAC stiffness Mean (SD)		62.8 (25.4)	33.0 (29.7)	N/A
WOMAC function Mean (SD)		59.8 (20.6)	34.0 (24.3)	N/A
NRS pain Mean (SD)		5.7 (2.3)	2.6 (2.4)	N/A
HADS Mean (SD)		12.6 (7.2)	9.6 (6.7)	N/A
MOAKS* N (%) BML	MOAKS=0 MOAKS=1 MOAKS=2 MOAKS=3	9 (14.1) 52 (81.3) 3 (4.6) 0 (0)	4 (57.1) 3 (42.9) 0 (0) 0 (0)	N/A
Synovitis/effusion N (%)	MOAKS=0 MOAKS=1 MOAKS=2 MOAKS=3	2 (3.1) 28 (43.8) 18 (28.1) 16 (25)	2 (28.6) 2 (28.6) 1 (14.2) 2 (28.6)	N/A
Cartilage damage N (%)	MOAKS=0 MOAKS=1 MOAKS=2 MOAKS=3	0 (0) 16 (25) 41 (64.1) 7 (10.9)	4 (57.1) 3 (42.9) 0 (0) 0 (0)	N/A
Clinical Management		Underwent knee replacement surgery	Medical management	Underwent other surgery

BML, bone marrow lesion; HADS, Hospital Anxiety and Depression Scale; MOAKS, MRI Knee Osteoarthritis Score; NRS, Numerical Rating Scale; OA, osteoarthritis; WOMAC, Western Ontario and McMaster Universities Osteoarthritis Index.

A mixture of OA participants was identified, and they were classified as severe or mild based on MRI. In the advanced OA group, 81.3% of participants had up to 33% of the bone volume (MOAKS score 1) forming a BML in at least one of the 21 measured regions, in addition to significant levels of synovitis and cartilage damage (table 1). MRI scans found BML areas to be invariably associated with regions of established cartilage damage, particularly in medial tibial regions, which were the focus of our tissue and microarray to maintain consistency of anatomical tissue lesions analysed. We found that 37.5% of grade 1 and 2 BML were in the medial tibial compartment, with 12.5% in the lateral tibial compartment. The remainder were distributed in the femur, trochlea and patella. For microarray, 50% samples were localised in the medial tibial compartment, 35.7% were found in the lateral tibial compartment and 14.2% crossed both tibial compartments.

Trends for WOMAC pain with individual MOAKS modalities showed higher WOMAC pain scores were associated with significantly greater BMLs in the advanced OA versus mild OA groups (see online supplementary figure S1). There was also a trend of increasing WOMAC pain with worsening MOAKS-scored synovitis, although these correlations did not reach statistical significance.

10.1136/annrheumdis-2017-211396.supp2 Supplementary file 2

**Figure 1:**
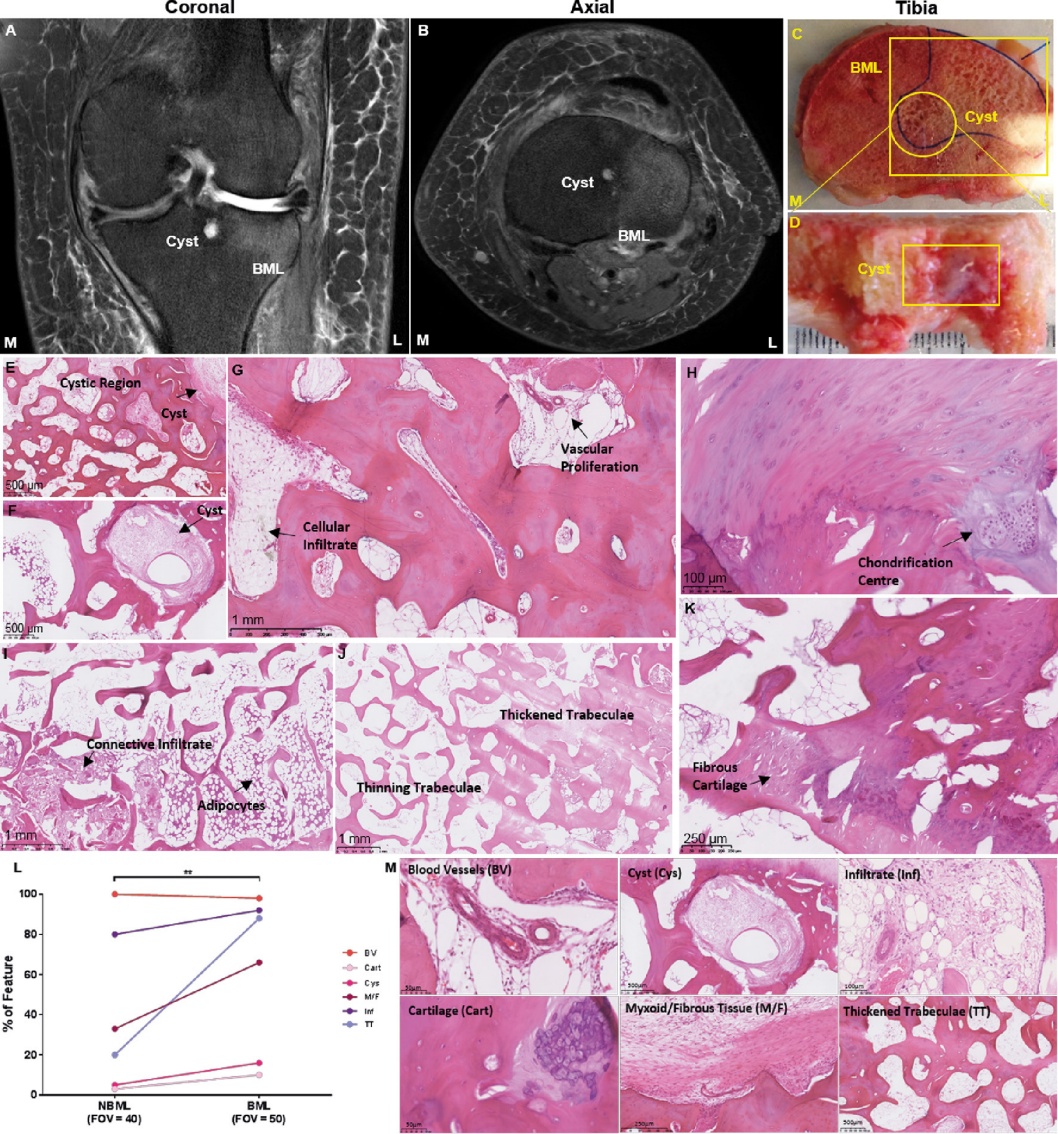
(A) Coronal plane of MRI scan visualising BML and associated cyst. (B) Axial plane of MRI scan presenting BML and associated cyst. (C) Macroscopic view of tibial BML and cystic area. (D) Image of cross section cut through BML and cyst localised by MRI revealing a gelatinous aggregate. (E) H&E staining of cystic region presenting cellular infiltrate in marrow spaces. (F) H&E staining of subchondral cyst forming. (G) H&E staining of BML region with vascular proliferation and cellular infiltration. (H) H&E staining of BML visualising a chondrification centre near the tidemark. (I) H&E staining of adipocyte in bone compartment with a soft tissue infiltrate working through osteoid network. (J) H&E staining of BML showing areas of thickened trabecular adjacent to thinning trabeculae. (K) H&E staining of BML demonstrating areas of fibrotic cartilage formation within the subchondral bone compartment. (L) Quantification of histology analysing 50 BML FOVs and 40 non-BML (NBML) FOVs for blood vessels (BV), cartilage within bone compartment (Cart), cysts (Cys), myxoid/fibrous tissue (M/F), cellular infiltrate (Inf) and trabecular thickening (TT) (n=4). A percentage for the presence of each histological feature was determined for each group. Significance was tested between the groups using Friedman test (*p<0.05). (M) Magnification of each histological change within the bone compartment: BV within subchondral bone, Cart within bone compartment with a chondrification centre, Cys within subchondral bone, M/F adjacent to subchondral bone, Inf within the osteoid network and TT. BML, bone marrow lesion; FOV, field of view.

Histological analysis showed most normal bone marrow was adipocytic with adipocytes being the primary bone lining cells (figure 1). The bone volume fraction was starkly reduced in BML areas, with marrow replaced by new blood vessels, dense fibrous connective tissue, hyaline cartilage and fibrocartilage. Areas of aggressive resorption were found at the periphery of BML zones alongside regions of cartilaginous aggregates found at least 2 mm deep to the articular surface embedded within the bone compartment. Regions of vascular proliferation with fibrocartilage were interspersed with areas of de novo cartilage formation. Other BML regions exhibited a cellular infiltrate working through the osteoid network. Histological quantification found the BML group had increased vascular proliferation, cellular infiltration and trabecular thickening when compared with the non-BML (NBML) group (p<0.05).

**Figure 2:**
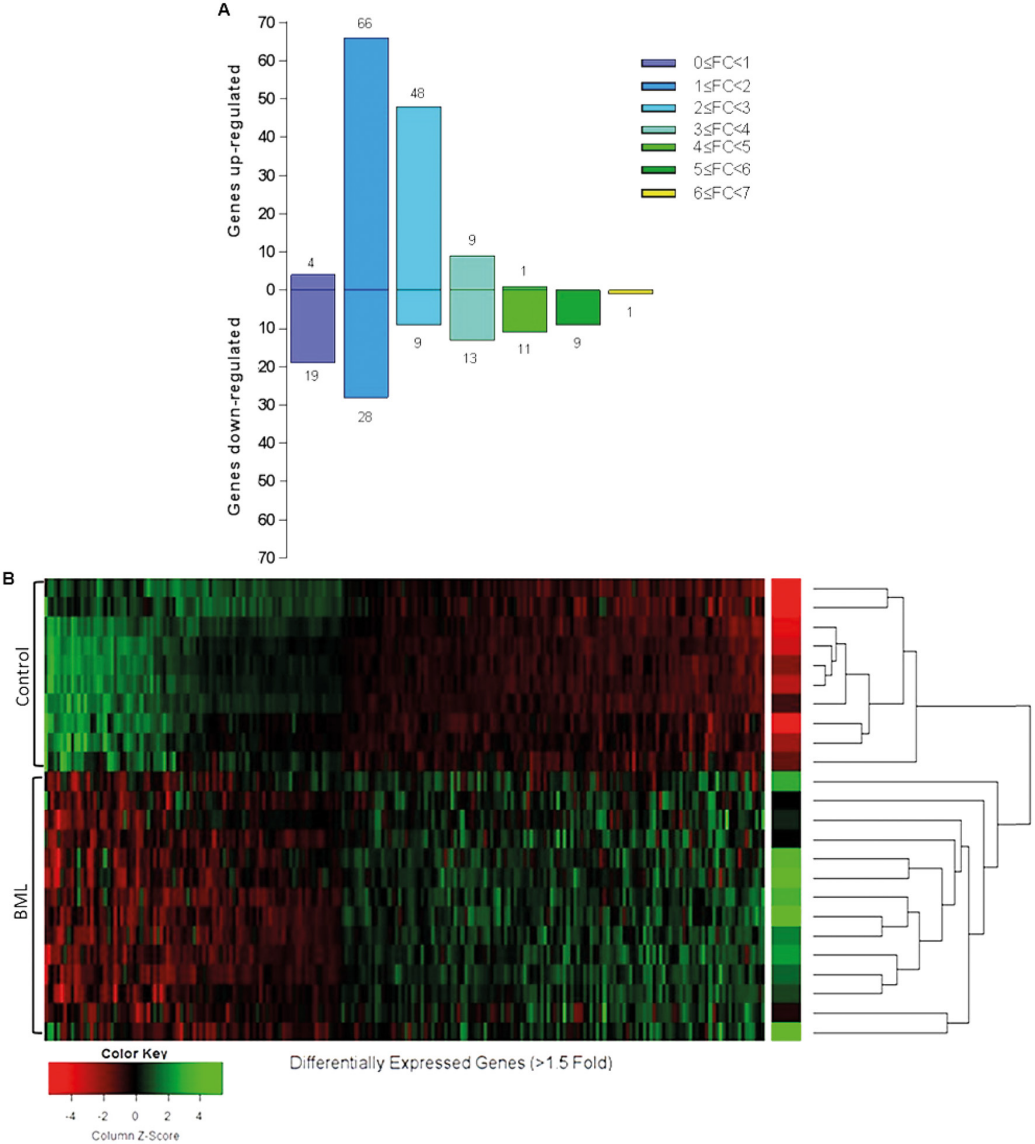
(A) Bar chart presenting the most significantly upregulated and downregulated entities by fold change (FC). One hundred twenty-eight entities were found to be upregulated and 90 were downregulated. The mean WOMAC pain score in the OA microarray group was 61.4, and all subjects in the OA array group had a MOAKS BML score of at least 1, with cartilage and synovitis scores of at least 2. (B) Pearson’s correlation hierarchical clustering of 218 genes clearly segregating the OA BML group from the control group.

**Figure 3:**
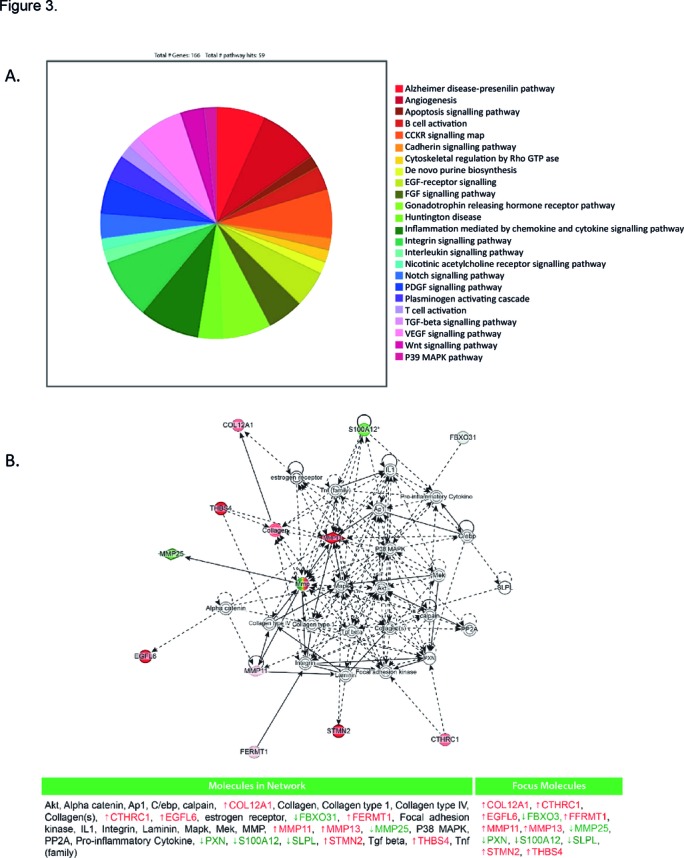
(A) Gene ontology analysis of 218 differentially expressed entities found 166 genes associated with 59 canonical pathways. Pie chart of the 24 predominant pathways identified. The main significant correlation for WOMAC pain with gene correlation was for MMP-13 (p<0.05). (B) Network analysis was performed on the differentially expressed genes by ingenuity pathway analysis (IPA). MMP-13, matrix metalloproteinase 13; WOMAC, Western Ontario and McMaster Universities Osteoarthritis Index.

Whole transcriptomic analysis identified 218 entities to be significantly differentially expressed between the OA BML and control bone samples (p<0.05) (figures 2 and 3). The most highly upregulated genes were stathmin 2 (*STMN2*), ATP-binding cassette protein, thrombospondin 4 *(THBS4)*, matrix metalloproteinase 13 (*MMP-13*) and chromosome 21 open reading frame, which are genes involved in diverse functions including bone remodelling, pain sensitisation and matrix turnover (see Discussion). The most downregulated genes included haemoglobin, S100 calcium binding protein A12, hemogen, proplatelet basic protein ((chemokine C-X-C) motif ligand 7) and delta amino levulinate synthase 2 (table 2 see online supplementary table S1 for full list).

10.1136/annrheumdis-2017-211396.supp3 Supplementary file 3

Among other significantly upregulated genes were the epidermal growth factor (EGF)-like domain (*EGFL6*), which is involved in cell adhesion, apoptosis and calcium binding; collagen type XVI (*COL16A1*) with functions in ECM organisation, cell adhesion and integrin-mediated signalling; and G protein coupled receptor (*GPR158*), which facilitates signal transduction and binds hormones/neurotransmitters and ATPase H+ transporting lysosomal (*ATP6V0D2*) gene expressed at axon termini and synaptic vesicles that is implicated in neuron projection. We also found upregulation of the DIRAS family, GTP-binding RAS-like 2 (*DIRAS2*) which is a Ras GTPase implicated in neurodegeneration. PC4 and SFRS1 interacting protein 1 (*PSIP1*) were also identified and are molecules involved in neuroepithelial stem cell differentiation, neurogenesis and apoptosis. Neuronal tyrosine phosphorylated phosphoinositide-3-kinase adaptor 2 (*NYAP2*) was also detected, which is a gene involved in neuronal development, interacting with WAVE1 proteins and is implicated in cytoskeletal modelling. We also found catenin (cadherin-associated protein) (*CTNND2*) upregulation, an adhesive junction associated protein implicated in bone, pain sensitisation, brain development and cancer formation.

Gene ontology analysis identified 166 of the 218 significantly differentially regulated entities to be associated with 59 canonical pathways. The angiogenic, Alzheimer disease-presenilin pathway, EGF/FGF/gonadotrophin signalling, inflammation mediated by chemokine and cytokine signalling with PDGF/Notch/vascular epidermal growth factor (VEGF) and Wnt signalling pathways were a few of which had the greatest number of entities related.

**Figure 4:**
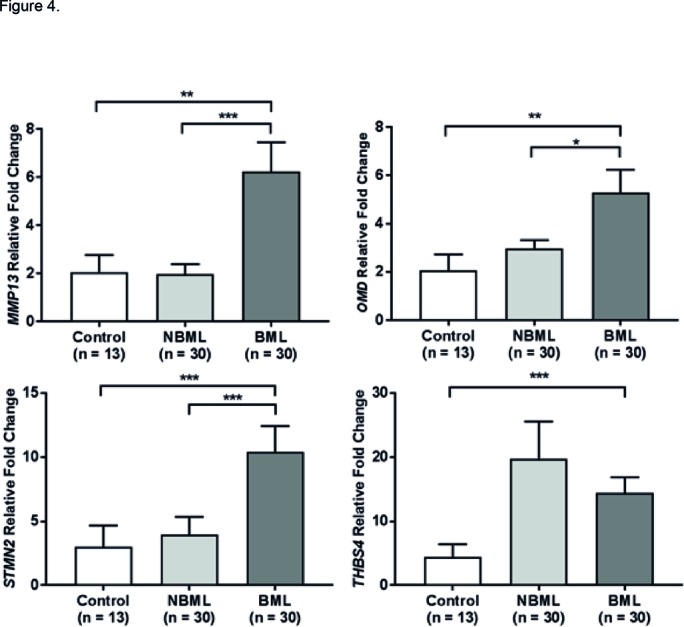
qPCR validation for stathmin 2 (*STMN2*), thrombospondin 4 (*THBS4*), matrix metalloproteinase 13 (*MMP-13*) and osteomodulin (*OMD*) of OA BML compared non-BML tissue and control bone. *STMN2*, *THBS4* and *MMP-13* were selected as they were among the most upregulated genes from the microarray. Osteomodulin was selected as a bone-specific marker as it is involved in bone homeostasis (**p<0.005, ***p<0.0005). BML, bone marrow lesion; NBML, non-bone marrow lesion; OA, osteoarthritis.

Quantitative polymerase chain reaction analysis confirmed *STMN2*, *MMP-13* and *THBS4* were significantly upregulated in BML regions compared with the control comparator group. *THBS4* and *STMN2* were the most highly upregulated genes between the BML and control bone groups (p<0.0001), reflecting comparable results to the microarray (figure 4). *MMP-13* and *STMN2* were upregulated within BML regions compared with NBML matched regions (p<0.0001). However *THBS4* was found to be most upregulated in the NBML compared with both BML and control groups. Serum STMN2 levels were not significantly increased in mild/ advanced OA groups compared with controls. Protein quantification of STMN2 in BML tissue found control bone to have higher presence of STMN2 compared with BML bone (p<0.0001). Functional significance of *MMP-13* protein activity, one of the highest array-expressed genes, found a significant increase in urine CTX-II levels, that is, cleavage products of type II collagen, in the advanced OA group compared with mild OA and control groups (p<0.001) (see online supplementary figure S2).

10.1136/annrheumdis-2017-211396.supp4 Supplementary file 4

**Table 2:** Summary of the top differentially expressed entities between the OA BML and non-OA control groups using whole transcriptomic analysis

**Accession no**	**Symbol**	**Entity name**	**↑↓**	**Abs FC**	**Log FC**	P Value*	**P Value**†
**NM_007029**	*STMN2*	Stathmin 2	Up	19.30	4.27	3.67 × 10^−6^	1.6 × 10^−6^
**NM_001163942**	*ABCB5*	ATP-binding cassette, sub-family B (MDR/TAP), member 5	Up	12.11	3.60	2.06 × 10^−6^	8.86 × 10^−7^
**NM_003248**	*THBS4*	Thrombospondin 4	Up	11.53	3.53	1.31 × 10^−4^	7.35 × 10^−5^
**NM_002427**	*MMP13*	Matrix Metallopeptidase 13 (collagenase 3)	Up	11.18	3.48	2.78 × 10^−5^	1.41 × 10^−5^
**NR_037585**	*C21orf37*	Chromosome 21 open reading frame 37	Up	9.32	3.22	3.64 × 10^−6^	1.65 × 10^−6^
**NM_001167890**	*EGFL6*	EGF-like-domain, multiple 6	Up	9.07	3.18	2.69 × 10^−5^	1.38 × 10^−5^
**NM_001856**	*COL16A1*	Collagen, type XVI, alpha 1	Up	8.25	3.04	1.8 × 10^−5^	9.08 × 10^−6^
**NM_020752**	*GPR158*	G protein-coupled receptor 158	Up	8.21	3.04	1.13 × 10^−4^	6.35 × 10^−5^
**NM_012093**	*AK5*	Adenylate kinase 5	Up	8.01	3.00	5.77 × 10^−6^	2.73 × 10^−6^
**NM_174858**	*AK5*	Adenylate kinase 5	Up	8.01	3.00	3.33 × 10^−5^	1.74 × 10^−5^
**NM_152565**	*ATP6V0D2*	ATPase, H+ transporting, lysosomal 38kDa, V0 subunit d2	Up	7.89	2.98	4.11 × 10^−6^	1.91 × 10^−6^
	*ALU2*	Alu 2 Element	Up	7.44	2.89	1.32 × 10^−6^	5.82 × 10^−7^
**NM_017594**	*DIRAS2*	DIRAS family, GTP-binding RAS-like 2	Up	7.14	2.84	2.8 × 10^−6^	1.29 × 10^−6^
**XR_245643**	*LOC101929504*	Uncharacterized LOC101929504	Up	7.02	2.81	3.79 × 10^−5^	2.02 × 10^−5^
**NM_021233**	*DNASE2B*	Deoxyribonuclease II beta	Up	7.02	2.81	1.55 × 10^−5^	7.86 × 10^−6^
**NM_014980**	*STXBP5L*	Syntaxin binding protein 5-like	Up	6.72	2.75	2.68 × 10^−6^	1.24 × 10^−6^
**NM_004789**	*LHX2*	LIM homeobox 2	Up	6.71	2.75	7.61 × 10^−5^	4.23 × 10^−5^
**NM_021144**	*PSIP1*	PC4 and SFRS1 interacting protein 1	Up	6.57	2.72	3.62 × 10^−6^	1.71 × 10^−6^
**NM_020864**	*NYAP2*	Neuronal tyrosine-phosphorylated phosphoinositide-3-kinase adaptor 2	Up	6.48	2.70	2.53 × 10^−5^	1.33 × 10^−5^
**NM_001332**	*CTNND2*	Catenin (cadherin-associated protein), delta 2	Up	6.36	2.67	6.52 × 10^−6^	3.19 × 10^−6^
**NM_032532**	*FNDC1*	Fibronectin type III domain containing 1	Up	6.09	2.61	7 × 10^−5^	3.91 × 10^−5^
**NM_001426**	*EN1*	Engrailed homeobox 1	Up	5.75	2.52	1.21 × 10^−6^	5.56 × 10^−7^
**NR_027054**	*MIR31HG*	MIR31 host gene (non-protein coding)	Up	5.64	2.50	1.21 × 10^−6^	1.03 × 10^−4^
	*XLOC_006820*		Up	5.48	2.45	9.05 × 10^−6^	4.6 × 10^−6^
**NM_014728**	*FRMPD4*	FERM and PDZ domain containing 4	Up	5.34	2.42	3.09 × 10^−5^	1.68 × 10^−5^
**TCONS_00014487**	*LOC101929450*	Uncharacterized LOC101929450	Up	5.33	2.41	1.31 × 10^−5^	6.78 × 10^−6^
**NM_022970**	*FGFR2*	Fibroblast growth factor receptor 2	Up	5.30	2.41	9.69 × 10^−6^	4.97 × 10^−6^
**NM_012152**	*LPAR3*	Lysophosphatidic acid receptor 3	Up	5.27	2.40	3.65 × 10^−5^	2 × 10^−5^
**NM_004370**	*COL12A1*	Collagen, type XII, alpha 1	Up	5.27	2.40	1.32 × 10^−6^	6.2 × 10^−7^
**BC043571**	*LOC613266*	Uncharacterized LOC613266	Up	5.09	2.35	1.2 × 10^−7^	5.25 × 10^−8^
**NM_000170**	*GLDC*	Glycine dehydrogenase (decarboxylating)	Up	5.00	2.32	6.11 × 10^−5^	3.46 × 10^−5^
**NM_031913**	*ESYT3*	Extended synaptotagmin-like protein 3	Up	5.00	2.32	3.61 × 10^−5^	1.99 × 10^−5^
	*ALU1*	Alu 1 Element	Down	−5.02	−2.33	3.17 × 10^−7^	1.44 × 10^−7^
**NM_025260**	*C6orf25*	Chromosome 6 open reading frame 25	Down	−5.82	−2.54	5.35 × 10^−6^	2.62 × 10^−6^
**NM_080429**	*AQP10*	Aquaporin 10	Down	−6.92	−2.79	6.26 × 10^-−7^	2.62 × 10^−6^
**NM_005306**	*FFAR2*	Free fatty acid receptor 2	Down	−7.29	−2.87	5.63 × 10^−5^	3.06 × 10^−5^
**AB305916**	*TRBV28*	T Cell Receptor Beta Variable 28	Down	−7.50	−2.91	3.35 × 10^−6^	1.55 × 10^−6^
**NM_000517**	*HBA2*	Hemoglobin, alpha 2	Down	−7.64	−2.93	7.61 × 10^−7^	3.25 × 10^−7^
	*XLOC_014512*		Down	−7.99	−3.00	2.74 × 10^−7^	1.1 × 10^−7^
**NM_000517**	*HBA2*	Hemoglobin, alpha 2	Down	−8.20	−3.04	5.59 × 10^−7^	2.33 × 10^−7^
**NM_016509**	*CLEC1B*	C-type lectin domain family 1, member B	Down	−8.24	−3.04	1.03 × 10^−4^	2.33 × 10^−7^
**NM_002620**	*PF4V1*	Platelet factor 4 variant 1	Down	−9.31	−3.22	2.34 × 10^−6^	1.04 × 10^−6^
**NM_022468**	*MMP25*	Matrix Metallopeptidase 25	Down	−9.33	−3.22	4.32 × 10^−5^	2.28 × 10^−5^
**NR_120522**	*LOC102724484*	Uncharacterized LOC102724484	Down	−10.04	−3.33	1.01 × 10^−4^	5.6 × 10^−5^
**NM_001136503**	*SMIM24*	Small integral membrane protein 24	Down	−10.29	−3.36	1.38 × 10^−5^	6.73 × 10^−6^
**NM_030773**	*TUBB1*	Tubulin, beta 1 class VI	Down	−12.37	−3.63	5.86 × 10^−7^	2.34 × 10^−7^
	*HSJ1167H4*		Down	−13.17	−3.72	3.71 × 10^−6^	1.65 × 10^−6^
**NR_001552**	*TTTY16*	Testis-specific transcript, Y-linked 16 (non-protein coding)	Down	−13.65	−3.77	6.28 × 10^−5^	3.34 × 10^−5^
**NR_047499**	*LINC00570*	Long intergenic non-protein coding RNA 570	Down	−14.00	−3.81	1.03 × 10^−4^	8.67 × 10^−5^
**NM_144673**	*CMTM2*	CKLF-like MARVEL transmembrane domain containing 2	Down	−14.25	−3.83	2.71 × 10^−5^	1.36 × 10^−5^
**NM_001557**	*CXCR2*	Chemokine (C-X-C motif) receptor 2	Down	−14.93	−3.90	9.27 × 10^−6^	4.34 × 10^−6^
**NM_000519**	*HBD*	Hemoglobin, delta	Down	−15.75	−3.98	7.89 × 10^−8^	2.74 × 10^−8^
**NM_002100**	*GYPB*	Glycophorin B (MNS blood group)	Down	−16.15	−4.01	1.03 × 10^−4^	1.43 × 10^−4^
**XM_005261527**	*SEC14L3*	SEC14-like 3 (S. cerevisiae)	Down	−16.65	−4.06	2.98 × 10^−5^	1.5 × 10^−5^
**AK128128**	*FLJ46249*		Down	−16.90	−4.08	6.19 × 10^−5^	3.27 × 10^−5^
**NM_016509**	*CLEC1B*	C-type lectin domain family 1, member B	Down	−17.06	−4.09	1.34 × 10^−5^	6.39 × 10^−6^
**NM_016509**	*CLEC1B*	C-type lectin domain family 1, member B	Down	−17.67	−4.14	4.83 × 10^−6^	2.15 × 10^−6^
**NM_002049**	*GATA1*	GATA binding protein 1 (globin transcription factor 1)	Down	−19.55	−4.29	7.87 × 10^−5^	4.21 × 10^−5^
**NM_005764**	*PDZK1IP1*	PDZK1 interacting protein 1	Down	−20.36	−4.35	7.59 × 10^−6^	3.47 × 10^−6^
**NM_006163**	*NFE2*	Nuclear factor, erythroid 2	Down	−22.54	−4.49	3.22 × 10^−5^	1.62 × 10^−5^
	*XLOC_013489*		Down	−23.69	−4.57	2.85 × 10^−5^	1.42 × 10^−5^
**NM_002619**	*PF4*	Platelet factor 4	Down	−31.42	−4.97	1.26 × 10^−7^	4.32 × 10^−8^
	*XLOC_000346*		Down	−31.94	−5.00	1.26 × 10^−7^	2.56 × 10^−5^
**NM_000032**	*ALAS2*	Aminolevulinate, delta-, synthase 2	Down	−33.49	−5.07	1.93 × 10^−5^	9.3 × 10^−6^
**NM_005980**	*S100P*	S100 calcium binding protein P	Down	−33.56	−5.07	1.11 × 10^−4^	6.06 × 10^−5^
**NM_005331**	*HBQ1*	Hemoglobin, theta 1	Down	−34.07	-5.09	3.58 × 10^−6^	1.53 × 10^−6^
**NM_002704**	*PPBP*	Pro-platelet basic protein (chemokine (C-X-C motif) ligand 7)	Down	−39.94	−5.32	4.11 × 10^−8^	1.3 × 10^−8^
**NM_000517**	*HBA2*	Hemoglobin, alpha 2	Down	−41.07	−5.36	2.47 × 10^−7^	8.77 × 10^−8^
**NM_001003938**	*HBM*	Hemoglobin, mu	Down	−45.11	−5.50	7.66 × 10^-5^	4.05 × 10^−5^
**NM_018437**	*HEMGN*	Hemogen	Down	−53.12	−5.73	1.89 × 10^−6^	7.66 × 10^−7^
**NM_005621**	*S100A12*	S100 calcium binding protein A12	Down	−56.95	−5.83	7.25 × 10^−5^	3.81 × 10^−5^
**NM_005621**	*S100A12*	S100 calcium binding protein A12	Down	−58.82	−5.88	4.6 × 10−^5^	2.34 × 10^−5^
**NM_000559**	*HBG1*	Hemoglobin, gamma A	Down	−88.82	−6.47	1.94 × 10^−6^	7.82 × 10^−7^

Symbol, Entity Symbol. ↑↓, Regulation. Abs FC, Absolute Fold Change. Log FC, Log transformed Fold Change.

*Adjusted Student T-test P value for microarray corrected for multiple testing by the Bonferroni FWER method.

†Adjusted Moderated T-test P value for microarray corrected for multiple testing by the Bonferroni FWER method.

## Discussion

BMLs have been well described by MRI in knee OA,^6 7 18^ but very little is known about their transcriptomic expression. To our knowledge, our study is the first to use a multimodal approach with MRI to locate knee OA BMLs, followed by detailed histological analysis and whole transcriptomic techniques for a multivariate interrogation of the changes seen within BMLs.

Bone marrow signal changes were first described on MRI by Wilson *et al* who used the term ‘bone marrow oedema’ to describe MRI findings in painful joints.^19^ Studies so far have focused on acquiring data from patients undergoing joint surgery of the knee and hip. Zanetti *et al* determined histologically that BMLs contained normal fatty marrow with marrow necrosis, necrotic or remodelled trabeculae, oedema and bone marrow bleeding.^20^ The same group matched MRI changes to BML abnormalities in participants undergoing TKR and found regions of normal tissue alongside bone marrow fibrosis, oedema and bleeding. In a hip and knee OA study, Hunter *et al* reported increased bone volume fraction but decreased tissue mineral density within BML using light microscopy.^21^ Samples from the lesion area showed increased trabecular thickness, with granulation, oedema, necrosis, fibrinoid deposition and hyperplasia of blood vessels. Taljanovic reported one of the largest histological studies of hip OA BML, where regions of fibrosis and microfracture formation at different stages of healing were observed.^22^ Leydet-Quilici *et al* also described oedema, necrosis and fibrosis within BML biopsies.^23^ Using MRI, Roemer *et al* previously demonstrated that progression of disease and the development of BMLs correlated with an increased risk of cartilage loss within the same subregion and that regions without BMLs are associated with decreased risk of cartilage loss,^24^ changes that our work supported. Carrino *et al*^25^ reported 87% of subchondral cysts were associated with BML abnormalities, which our analysis confirmed on MRI and by histology. In comparison with other studies, our detailed MRI matching with histological techniques allowed improved visualisation of BMLs, with direct observation of areas appearing as BML-associated cystic structures on MRI and transcriptomic expression. We found higher WOMAC pain scores with greater MOAKS-measured cartilage damage, as suggested by previous studies.^7^

In our study, cystic BML areas were surrounded by regions of fibrosis, infiltration by inflammatory cells and vascular proliferation. Previous hypotheses that BMLs could be precystic but that not all BMLs become cystic is also supported by our histological findings, where we observed cystic structures within the areas defined as cysts using MRI, and also adjacent to areas of fibrocartilage, vascular proliferation, chondrogenesis and amorphous tissue deposition. We observed new cartilage forming deep within the subchondral bone compartment. The new cartilage tissue within the BML could be arising from mesenchymal stem cells (MSCs) in the marrow, which is seen by other groups.^26^ Campbell *et al* reported an altered phenotype of MSCs in hip OA BMLs, showing BML-derived MSCs undergo osteochondral angiogenesis and have lower proliferation and mineralisation capacities.^27^

From our microarray, the highest upregulated gene was *STMN2*, a phosphoprotein involved in regulating microtubule function, responsiveness to nerve growth factor (NGF), neuronal growth and osteogenesis.^28^ Upregulation of *STMN2* within BML could lead to new neuronal structures and expansion of the BML in OA, thereby causing pain.^29^ Stathmin 2 protein expression was higher in normal than BML bone, which could reflect increased stathmin 2 turnover in OA BMLs.

We also identified neuronal markers including thrombospondin 4 (*THBS4*), implicated in the inflammatory response to Central Nervous system (CNS) injury, presynaptic hypersensitivity and neuropathic pain states.^30^ In animal models of pain sensitisation, *THBS4* levels are increased locally in dorsal root ganglion neurons and contribute to pain behaviour, which can be inhibited by the calcium channel modulator gabapentin.^31^

Other upregulated genes involved in neuronal morphogenesis included *ATP6V0D2*, *PSIP1*, *NYAP2*, FERM and PDZ containing 4 (*FRMPD4*), implicated in CNS development and pain states.^32 33^ ECM genes were also represented in the array, including *MMP-13* and collagens, *COL16A1*, fibronectins and growth factors, which are known to be bound within the ECM.^34^

Our data demonstrate that BMLs are regions of high metabolic activity with increased cell turnover, bone remodelling, neuronal and inflammatory gene signatures. Gene ontological analysis revealed canonical pathways involved in chemokine, integrin and cytokine signalling. We found neurodevelopment and pain pathway signalling represented by the Alzheimer’s, Notch, catenin, Wnt pathways alongside VEGF and angiogenic pathway expression. Work by Hopwood *et al*^35^ and Chou *et al*^36^ analysing the gene expression profile of OA bone also found expression of bone remodelling signalling pathways including Wnt, transforming growth factor and bone morphogenic protein and bone remodelling molecules such as periostin and leptin. Kusumbe *et al* described how growth of blood vessels in bone and osteogenesis are coupled, proposing that type H endothelial cells mediate local growth of the vasculature and provide specific signals for perivascular osteoprogenitors.^37^ The same group reported that endothelial Notch activity promotes angiogenesis and osteogenesis in bone.^38^ We also demonstrated *OMD* in our BML tissue: Ninomiya *et al* showed that osteoclast activity induces *OMD* expression in bone, suggesting BMLs represent areas of active bone remodelling.^39^

The expression of both osteogenic and angiogenic genes along with the tissue changes we identified may suggest that vascular proliferation and bone formation are likely to be coupled in BML formation. Since blood vessels are formed within neurovascular bundles, it is likely that increased neuronal pathway gene expression including *STMN2*, *THBS4*, *PSIP1*, *NYAP2* and catenin, which were among some of the most highly expressed genes from our BML analysis, are implicated in neural pathway development, new nerve formation and pain mediation in BML tissue.

Our array also identified molecules within the Wnt signalling pathway, including catenin. Other studies have demonstrated a critical role for Wnt signalling in the production and persistence of neuropathic pain after nerve injury and bone cancer.^40^ Rodent models show that in nerve injury and bone cancer pain models, respectively, Wnt signalling is activated, which may contribute to pain by regulating pro-inflammatory cytokines interleukin-18 and tumour necrosis factor-alpha, as well as NR2B and subsequent Ca2+-dependent signals in the dorsal horn. We found a high representation of the inflammatory chemokines and cytokine signalling; other groups have also identified chemokines in OA pain, for example, *CCR2* was recently reported to mediate pain in a murine model of OA.^41^ Our data suggest that chemokine pathway molecules could be pain sensitisers in BMLs. Walsh *et al* showed that OA neurovascular changes at the osteochondral junction, including vessels and both sensory and sympathetic nerves breaching the tidemark, could possibly be a source of joint pain.^42^ The genes we have identified in our BML transcriptome support the hypothesis of neurovascular gene upregulation in BML tissue.

One of our most highly expressed genes was *MMP-13*, an enzyme expressed in cartilage, involved in regulating ECM turnover and cartilage destruction in OA.^43^ Our data showed that type II collagen degradation products were increased in urine from our advanced OA population. The de novo cartilage formation observed within BMLs, coupled with the increased transcriptomic expression of *MMP-13* observed using microarray and the detection of MMP-13 cleavage products, could suggest recapitulation of the embryonic bone development phenotype within OA BML regions.

Limitations of our study included the sample size for microarray, which although on a standard format of 24 samples, will benefit from larger studies. Future work for protein evaluation of the genes identified is needed, investigating which cells within BMLs are responsible for producing the genes identified and how BMLs develop with respect to the pathological changes identified in OA over time. Although we did not identify NGF, we found genes in neurotrophin pathways, including stathmin 2, which increases responsiveness to NGF,^28^ syntaxin, which regulates brain-derived neurotrophic factor^44^ and pituitary adenylate cyclase-activation polypeptide, implicated in neuronal development.^45^

In conclusion, our work demonstrates that BMLs are regions of high metabolic activity, with expression of genes involved in neuronal development, pain, ECM turnover, cartilage/bone formation and angiogenesis. Our findings contribute to understanding of OA pathogenesis and could help lead to the development of new diagnostic tools and future therapies for this most common arthritic disease.
