# Supplementary material for: Microarray analysis of bone marrow lesions in osteoarthritis demonstrates upregulation of genes implicated in osteochondral turnover, neurogenesis and inflammation
Source: Ann Rheum Dis. 2017 Jul 13;76(10):1764–73. doi: 10.1136/annrheumdis-2017-211396 (PMC5629942; doi:10.1136/annrheumdis-2017-211396)
Supplement: Supplementary file 1 [file annrheumdis-2017-211396supp001.docx]

**Supplementary methods**

**Study consent, control participants and exclusion criteria.**

Subjects were consented by a research associate independent of the treating physicians, for knee MRI of the target joint and waste joint tissue collection at TKR. Participants without knee OA were recruited as control subjects aged 35-90 years. Exclusion was made for other diagnoses e.g. rheumatoid arthritis, systemic lupus erythematosus, fibromyalgia, pregnancy, regular use of bisphosphonates, steroids or hormone replacement therapy within the last 6 months, history of clinically-diagnosed depression or anxiety and other recent surgery.

**Magnetic Resonance Imaging**

A Philips 3T MRI scanner acquired images from participants within six weeks before TKR to ensure the visualisation of structural changes, including BML, synovitis and cartilage damage. Multimodal MRI scans lasted 30 minutes and included scout images, T2-weighted imaging for lesion detection, 3D T1-weighted imaging for delineating knee structures, with T2 fat suppression for BML visualisation. All data were acquired using protocols that complied with scanner safety procedures and full CE-marking in place, with adherence to all contra-indications to MRI scanning. A dedicated 8 channel knee coil was used. Pulse sequences were sagittal, coronal and axial intermediate-weighted images with TE 30 ms, TR 500 ms and SPAIR fat-saturation and sagittal T1-weighted with TE 15 ms, TR 600 ms. _._Radiographic changes were evaluated independently for anonymised scans using the validated MRI Knee Osteoarthritis Score (MOAKS) (18) by two consultant radiologists (VE and CH) and consensus scores reached. For both BML size, cartilage damage and synovitis, a mean of the consensus scores for all of the regions was calculated for each participant.

**Microarray and QPCR methods**

The aim of the transcriptomic analysis was to select specific sites of OA BML pathology for analysis. As the largest number of significant lesions were observed in the tibial compartment, this site was selected for OA BML evaluation from consecutively enrolled participants. The control bone comparator group were distinct participants who were undergoing surgery following trauma, e.g. amputation, fracture correction or trochleoplasty, with no clinical or radiographic arthritis.

***Region of interest localisation and RNA isolation.*** MR images were viewed as DICOM files using an image processing and analysis software (ImageJ, NIH, Wayne Rasband, 1997). Axial image dimensions were set as: pixel width 0.25mm and pixel height: 0.2mm on ImageJ then scaled at 160mm x 160mm in line with the field of view. A 2cm x 2cm grid was drawn over the axial images and the MOAKS scoring was used to confirm the location of the lesions. The tibial plateau was placed over a grid of 2cm x 2cm with 1mm increments. This was used in line with the scaled axial images to measure and locate the BMLs. Once the lesions were located a region of bone of approximate equivalence that was macroscopically normal in appearance was taken approximately ≥5cm lateral to the lesion to use as the non-BML (NBML) control sample and both regions were dissected using a hack saw. The samples were washed in PBS and divided equally into two: one part stored at -80ºC for gene expression studies and the other fixed in 10% (v/v) natural buffered formalin (VWR, Leicestershire, UK) for histological analysis. A region of bone that was macroscopically normal in appearance was taken from the tissue control comparator bone and was dissected of all soft tissue and cartilage, washed in PBS and stored for future use at -80ºC.

***RNA isolation and Whole Transcriptomic Analysis.*** Total RNA was isolated from approximately 200mg of bone tissue using Qiazol and the RNeasy Mini Kit (Qiagen, Crawley, UK) according to manufacturer’s instructions (14). Total RNA was measured via spectrophotometry using a Nanodrop 1000 (Thermo Scientific, Hertfordshire, UK). Integrity and purity of the RNA was assessed via microfluidic electrophoretic technology using an Agilent 2100 bioanalyzer (RIN 6.5-8) (Agilent Technologies, Santa Clara, CA, US). Total RNA (200ng) isolated from bone tissue, from 10 healthy controls and 14 OA patients with extensive BMLs, were used for one round of cRNA synthesis and amplification using a LowInput QuickAmp Labelling Kit with the addition of positive control transcripts for monitoring the Agilent One Color Gene Expression microarray workflow using an RNA Spike In Kit – One Color (Agilent Technologies, Santa Clara, CA, USA). The Cyanine 3-labeled cRNA was quantified via spectrophotometry using a Nanodrop 1000 and purified using the RNeasy Mini Kit. The linearly amplified Cyanine 3-labeled cRNA samples (600ng) were hybridized to Agilent whole human genome 60k microarray chips and washed using buffers from the Gene Expression Wash Pack (Agilent Technologies, USA). Microarray chips were scanned using an Agilent SureScan High-Resolution DNA Microarray Scanner and Feature extraction performed using the Feature Extraction Software (version 11.5.1.1). Array signal intensities were analysed by the Agilent Gene-Spring GX software (version 11.5). The dataset was normalized by quantile normalization. Significant differentially expressed entities between bone samples from healthy controls and OA patients were selected using a union of a student’s and moderated t-test corrected for multiple comparisons with the Bonferroni FWER correction (P <0.05). Probes with a threshold of ≥1.5 fold-change were selected and these entities were additionally analysed by the hierarchical clustering method, with a Pearson correlation coefficient algorithm and an average linkage method. Significant differentially expressed gene IDs were uploaded onto PANTHER pathway analysis (PANTHER Classification Systems 10.0; [www.pantherdb.org](http://www.pantherdb.org)) for functional classification. The online resource includes functional annotations from the GO Phylogenetic Annotation project for the biological interpretation of large-scale experimental datasets (15, 16). The gene IDs were also loaded into Ingenuity Pathway Analysis (Ingenuity Systems; www.ingenuity.com) to identify functional annotations and predict biological interactions. The biological interaction scores were defined by the IPA statistical algorithm: based on the z-score and P-value, which were calculated by the IPA regulation z-score algorithm and the Fischer's exact test. A positive or negative z-score of more than 1.5 or less than 1.5, and P-value less than 0.05 (-log10 ≥1.5) indicates a significant biological function and predicts that the biological process or condition is leaning towards an increase (z-score ≥1.5) or decrease (z-score ≤ -1.5). P values were corrected for multiple comparisons with the Benjamini-Hochberg test.

***Quantitative Reverse-Transcription PCR Validation.*** To validate the microarray analysis quantitative polymerase chain reaction (qPCR) was performed for the most significantly upregulated genes. The genes of interest (GOI) were identified for verification on the basis of significant fold-change (≥1.5; P <0.05) between the OA BML, OA NBML and control groups. Expression of matrix metalloproteinase 13 (*MMP13*)*,* stathmin 2 (*STMN2*) and thrombospondin 4 (*THBS4*) was analysed using osteomodulin (*OMD*) a bone turnover gene expressed in osteoblasts and osteoclasts as a positive tissue control. To select a fixed group of reference genes the geNorm reference gene selection kit (PrimerDesign, Southampton, UK) was used. The kit incorporates 12 of the most notably cited candidate reference genes for which qPCR was used to measure expression in disease and non-disease tissue. The geNorm Biogazelle qbase PLUS software was used to analyse the data resulting in a list of the most optimal reference genes for accurate normalisation dependent on the stability of their expression. ATP Synthase, H+ Transporting, Mitochondrial F1 (*ATP5B)*, Cytochrome C1 (*CYC1*), Eukaryotic Translation Initiation Factor 4A2 (*EIF4A2*) and Succinate Dehydrogenase Complex Flavoprotein Subunit A (*SDHA*) were selected as endogenous reference genes. BML regions were analysed in 30 BML and 30 NBML matched bone tissue samples using 13 healthy control bone samples for the control comparator group. Total RNA from each region was reverse transcribed into 1µg of cDNA using Superscript II reverse transcriptase (Invitrogen, Paisley, UK). RNA samples below 100ng/µl were extracted, pooled and concentrated using a SpeedVac™ Concentrator (Savant SPD131DDA, Thermo Fisher Scientific, MA, USA). The qPCR was performed using GoTaq SYBR Green florescence system (Promega, Southampton, UK) according to the manufacturer's instructions. The qPCR reactions were performed on the Bio-Rad CFX96 or CFX384 Real-Time PCR Detection System (Bio-Rad Laboratories, Hertfordshire, UK) and the fluorescent signal intensity was analysed by CFX Manager Software (Bio-Rad Laboratories, Hertfordshire, UK). Two-tailed unpaired Mann Whitney U statistical testing was performed to evaluate statistically significant differences in gene expression levels between groups.

Primer sequences used are summarised below

**Histology**

Four patient samples were consecutively selected and specimens were fixed in 10% (v/v) neutral buffered formalin for 24 hours then decalcified using formic acid containing 40% (v/v) formalin. Specimens were placed in the decalcification solution at 20 times the tissue volume for up to 14 days at room temperature. Samples were dehydrated in graded alcohol series and paraffin embedded before being sectioned using a microtome at 5µm (Leica RM2255, Milton Keynes, UK). Sections were stained with haematoxylin and eosin (H&E) (Leica, Milton Keynes, UK). Slides were scanned using a NanoZoomer 2.0-RS Digital Scanner (Hamamatsu, Hertfordshire, UK) and visualised using the NanoZoomer Digital Pathology v2.0 software (Hamamatsu, Hertfordshire, UK). The scans were used to analyse 50 BML, 10 Cyst and 40 non-BML (NBML) fields of view (FOV) for the presence of blood vessels (BV), cartilage within the bone compartment (Cart), cysts (Cys), myxoid/fibrous tissue (M/F), cellular infiltrate (Inf) and trabecular thickening (TT). A percentage for the presence of each histological feature was determined for each group. Significance was tested between the two groups using the Friedman test (P<0.05).

**Enzyme-Linked ImmunoSorbent Assay**

***CTX-II ELISA.*** Detection of C-terminal telopeptides of type II collagen cleavage products was conducted using the Urine Cartilaps (CTX-II) EIA (Immunodiagnostic Systems) as previously described (17). Urine samples collected at the first visit were sampled for type II collagen cleavage products. Matched urine samples were tested for creatinine for normalisation. Absorbance of each well was read at 450 nm.

***STMN2 ELISA.*** Quantitative determination of STMN2 in human serum from 17 end-stage OA, 17 early OA and 7 healthy controls was performed using the US Biological STMN2 BioAssay ELISA Kit for human samples (United States Biological, Salem, MA, USA) adhering to manufacturer instructions. The ELISA kit employs the competitive enzyme-linked immunoassay technique with a colorimetric detection method measured at 450nm on a Molecular Devices Spectra Max 340 microplate reader (Molecular Devices, Berkshire, UK). The intensity of the absorbance is inversely proportional to the concentration of STMN2 present within the serum sample or standards. A standard curve was plotted relating the absorbance (OD) to the concentration of the standards (ng/ml). The STMN2 concentration in each sample was interpolated from the standard curve and results plotted on GraphPad Prism 7.01 (GraphPad Prism, San Diego, CA, USA). Kruskal-Wallis statistical testing was performed to evaluate statistically significant differences in STMN2 levels between groups

**Quantitative Western Blot Analysis**

For the quantitative detection of STMN2 within the bone samples 26 BML bone and 10 control bone samples were homogenised in RIPA buffer with protein inhibitors (PMSF, aprotinin and 1mM sodium orthovanadate) using a Fast Prep homogeniser for 3 bursts at 20 seconds, sonicated 3 x 5 seconds at 60% amplitude and clarified by centrifugation for 10 minutes. Appropriate dilutions of the supernatant were used in a Bradford protein assay (Sigma – Aldrich, MO, USA). Samples were denatured in 5X sample buffer at 99ºC and 50µg of protein loaded and run on SDS-Tris gels. All gels were run using the Bio-Rad Mini PROTEAN Tetra electrophoretic tank system run at a constant current (60mA) for 1-2 hours. The Gels were transferred onto PVDF using the Bio-Rad Mini-PROTEAN transfer system at a constant current (300mA) for 1 hour (Bio-Rad Laboratories, Hertfordshire, UK). Membranes were incubated with Odyssey PBS blocking buffer (1:1 dilution) (LI-COR Biosciences, NE, USA) prior to incubation with rabbit polyclonal antibody directed against STMN2 (1:500, Thermo Fischer Scientific, MA, USA, #720178) and mouse monoclonal anti-β-actin (1:2000, Sigma-Aldrich MO, USA, #A2228) made up in LiCor blocking buffer plus 0.2% (v/v) Tween^®^20 overnight at 4°C. Goat anti-rabbit IgG (H+L) Alexa Fluor^®^ 700 and donkey anti-mouse IgG Alexa Fluor^®^ 790 (1:10,000, Thermo Fischer Scientific, MA, USA, #A21038, #A11371) made up in LiCor blocking buffer plus 0.2% (v/v) Tween^®^ 20 were applied for 60 minutes at room temperature prior to washing with PBS + 0.1% (v/v) Tween^®^20. Visualisation and quantification of the blots were carried out using the LI-COR Odyssey^®^ scanner and software (LI-COR Biosciences). Normalised signal intensity values were obtained by subtracting the background and dividing the protein of interest (STMN2) densities by the relative normalising control (β-actin) values. The values were log transformed and evaluated for statistical significance using a two-tailed unpaired t-test.
