# Supplementary figures and images for: Microarray analysis of bone marrow lesions in osteoarthritis demonstrates upregulation of genes implicated in osteochondral turnover, neurogenesis and inflammation
Source: Ann Rheum Dis. 2017 Jul 13;76(10):1764–73. doi: 10.1136/annrheumdis-2017-211396 (PMC5629942; doi:10.1136/annrheumdis-2017-211396)

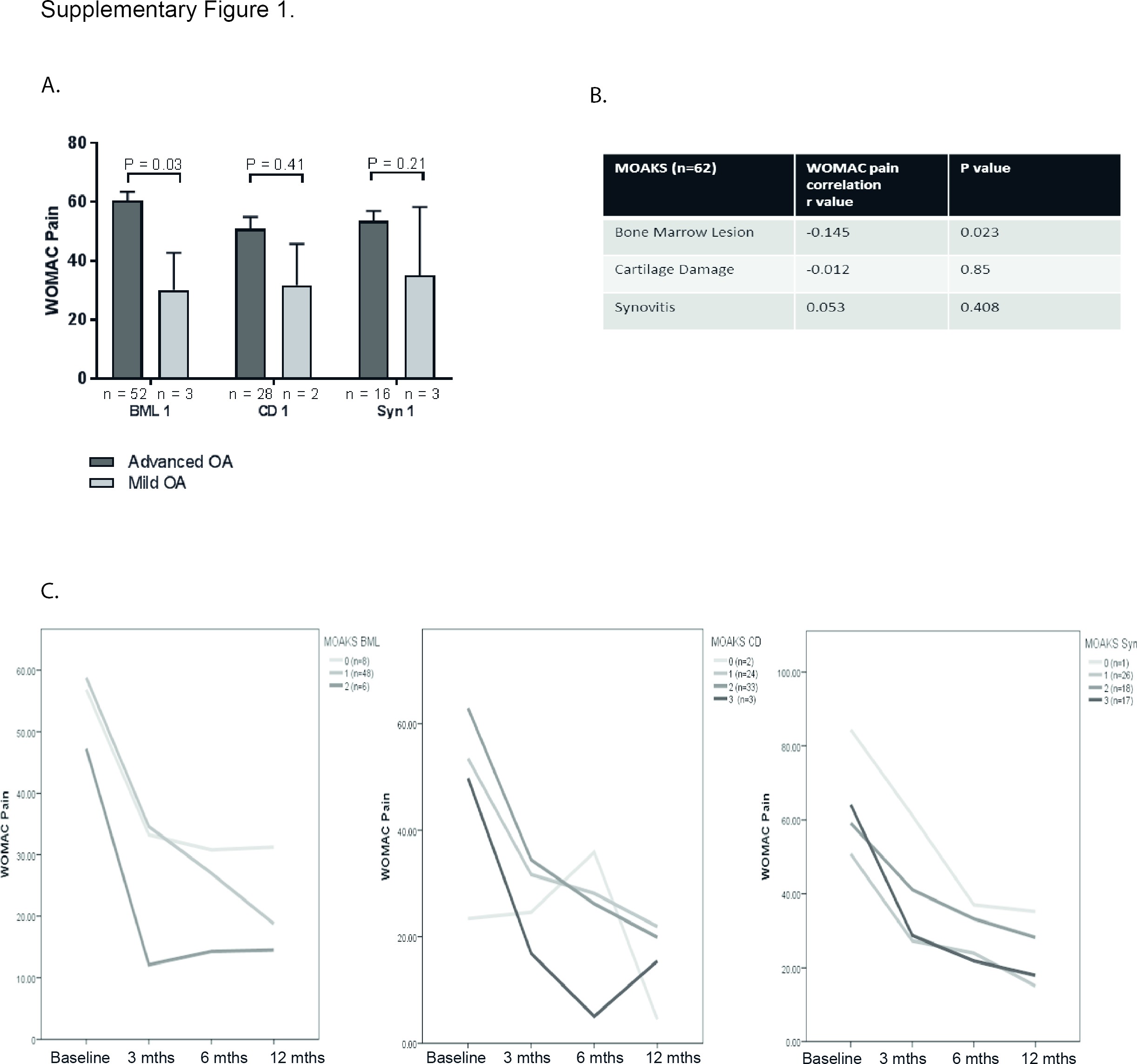

Supplement: Supplementary file 2 [file annrheumdis-2017-211396supp002.jpg]

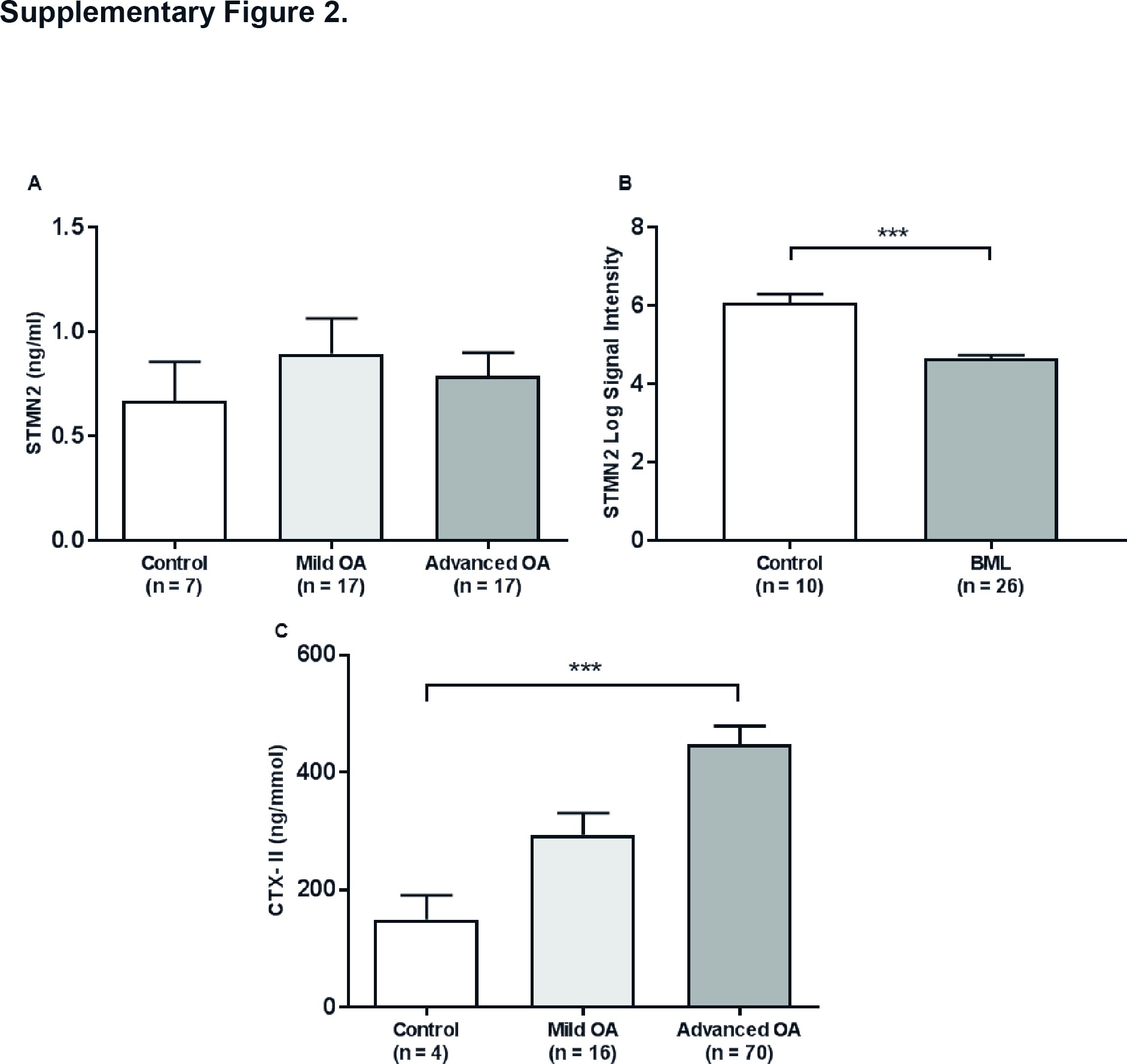

Supplement: Supplementary file 4 [file annrheumdis-2017-211396supp004.jpg]
