# Supplementary material for: Microarray analysis of bone marrow lesions in osteoarthritis demonstrates upregulation of genes implicated in osteochondral turnover, neurogenesis and inflammation
Source: Ann Rheum Dis. 2017 Jul 13;76(10):1764–73. doi: 10.1136/annrheumdis-2017-211396 (PMC5629942; doi:10.1136/annrheumdis-2017-211396)
Supplement: Supplementary file 3 [file annrheumdis-2017-211396supp003.docx]

| Accession # | Symbol | Entity Name | ↑↓ | Abs FC | Log FC | P Value^a^ | P Value^b^ |
| --- | --- | --- | --- | --- | --- | --- | --- |
| NM_007029 | *STMN2* | Stathmin 2 | Up | 19.30 | 4.27 | 3.67 x 10^-6^ | 1.6 x 10^-6^ |
| NM_001163942 | *ABCB5* | ATP-binding cassette, sub-family B (MDR/TAP), member 5 | Up | 12.11 | 3.60 | 2.06 x 10^-6^ | 8.86 x 10^-7^ |
| NM_003248 | *THBS4* | Thrombospondin 4 | Up | 11.53 | 3.53 | 1.31 x 10^-4^ | 7.35 x 10^-5^ |
| NM_002427 | *MMP13* | Matrix Metallopeptidase 13 (collagenase 3) | Up | 11.18 | 3.48 | 2.78 x 10^-5^ | 1.41 x 10^-5^ |
| NR_037585 | *C21orf37* | Chromosome 21 open reading frame 37 | Up | 9.32 | 3.22 | 3.64 x 10^-6^ | 1.65 x 10^-6^ |
| NM_001167890 | *EGFL6* | EGF-like-domain, multiple 6 | Up | 9.07 | 3.18 | 2.69 x 10^-5^ | 1.38 x 10^-5^ |
| NM_001856 | *COL16A1* | Collagen, type XVI, alpha 1 | Up | 8.25 | 3.04 | 1.8 x 10^-5^ | 9.08 x 10^-6^ |
| NM_020752 | *GPR158* | G protein-coupled receptor 158 | Up | 8.21 | 3.04 | 1.13 x 10^-4^ | 6.35 x 10^-5^ |
| NM_012093 | *AK5* | Adenylate kinase 5 | Up | 8.01 | 3.00 | 5.77 x 10^-6^ | 2.73 x 10^-6^ |
| NM_174858 | *AK5* | Adenylate kinase 5 | Up | 8.01 | 3.00 | 3.33 x 10^-5^ | 1.74 x 10^-5^ |
| NM_152565 | *ATP6V0D2* | ATPase, H+ transporting, lysosomal 38kDa, V0 subunit d2 | Up | 7.89 | 2.98 | 4.11 x 10^-6^ | 1.91 x 10^-6^ |
|  | *ALU2* | Alu 2 Element | Up | 7.44 | 2.89 | 1.32 x 10^-6^ | 5.82 x 10^-7^ |
| NM_017594 | *DIRAS2* | DIRAS family, GTP-binding RAS-like 2 | Up | 7.14 | 2.84 | 2.8 x 10^-6^ | 1.29 x 10^-6^ |
| XR_245643 | *LOC101929504* | Uncharacterized LOC101929504 | Up | 7.02 | 2.81 | 3.79 x 10^-5^ | 2.02 x 10^-5^ |
| NM_021233 | *DNASE2B* | Deoxyribonuclease II beta | Up | 7.02 | 2.81 | 1.55 x 10^-5^ | 7.86 x 10^-6^ |
| NM_014980 | *STXBP5L* | Syntaxin binding protein 5-like | Up | 6.72 | 2.75 | 2.68 x 10^-6^ | 1.24 x 10^-6^ |
| NM_004789 | *LHX2* | LIM homeobox 2 | Up | 6.71 | 2.75 | 7.61 x 10^-5^ | 4.23 x 10^-5^ |
| NM_021144 | *PSIP1* | PC4 and SFRS1 interacting protein 1 | Up | 6.57 | 2.72 | 3.62 x 10^-6^ | 1.71 x 10^-6^ |
| NM_020864 | *NYAP2* | Neuronal tyrosine-phosphorylated phosphoinositide-3-kinase adaptor 2 | Up | 6.48 | 2.70 | 2.53 x 10^-5^ | 1.33 x 10^-5^ |
| NM_001332 | *CTNND2* | Catenin (cadherin-associated protein), delta 2 | Up | 6.36 | 2.67 | 6.52 x 10^-6^ | 3.19 x 10^-6^ |
| NM_032532 | *FNDC1* | Fibronectin type III domain containing 1 | Up | 6.09 | 2.61 | 7 x 10^-5^ | 3.91 x 10^-5^ |
| NM_001426 | *EN1* | Engrailed homeobox 1 | Up | 5.75 | 2.52 | 1.21 x 10^-6^ | 5.56 x 10^-7^ |
| NR_027054 | *MIR31HG* | MIR31 host gene (non-protein coding) | Up | 5.64 | 2.50 | 1.21 x 10^-6^ | 1.03 x 10^-4^ |
|  | *XLOC_006820* |  | Up | 5.48 | 2.45 | 9.05 x 10^-6^ | 4.6 x 10^-6^ |
| NM_014728 | *FRMPD4* | FERM and PDZ domain containing 4 | Up | 5.34 | 2.42 | 3.09 x 10^-5^ | 1.68 x 10^-5^ |
| TCONS_00014487 | *LOC101929450* | Uncharacterized LOC101929450 | Up | 5.33 | 2.41 | 1.31 x 10^-5^ | 6.78 x 10^-6^ |
| NM_022970 | *FGFR2* | Fibroblast growth factor receptor 2 | Up | 5.30 | 2.41 | 9.69 x 10^-6^ | 4.97 x 10^-6^ |
| NM_012152 | *LPAR3* | Lysophosphatidic acid receptor 3 | Up | 5.27 | 2.40 | 3.65 x 10^-5^ | 2 x 10^-5^ |
| NM_004370 | *COL12A1* | Collagen, type XII, alpha 1 | Up | 5.27 | 2.40 | 1.32 x 10^-6^ | 6.2 x 10^-7^ |
| BC043571 | *LOC613266* | Uncharacterized LOC613266 | Up | 5.09 | 2.35 | 1.2 x 10^-7^ | 5.25 x 10^-8^ |
| NM_000170 | *GLDC* | Glycine dehydrogenase (decarboxylating) | Up | 5.00 | 2.32 | 6.11 x 10^-5^ | 3.46 x 10^-5^ |
| NM_031913 | *ESYT3* | Extended synaptotagmin-like protein 3 | Up | 5.00 | 2.32 | 3.61 x 10^-5^ | 1.99 x 10^-5^ |
| NM_012194 | *KIAA1549L* | KIAA1549-like | Up | 4.90 | 2.29 | 1.69 x 10^-5^ | 9.02 x 10^-6^ |
| NM_001287763 | *NSG1* | Neuron specific gene family member 1 | Up | 4.84 | 2.27 | 1.3 x 10^-4^ | 7.67 x 10^-5^ |
| NM_207338 | *LCTL* | Lactase-like | Up | 4.78 | 2.26 | 7.91 x 10^-6^ | 4.10 x 10^-6^ |
| NM_001004342 | *TRIM67* | Tripartite motif containing 67 | Up | 4.78 | 2.26 | 3.19 x 10^-5^ | 1.76 x 10^-5^ |
| NM_006240 | *PPEF1* | Protein phosphatase, EF-hand calcium binding domain 1 | Up | 4.76 | 2.25 | 1.01 x 10^-4^ | 1.01 x 10^-4^ |
| NM_001935 | *DPP4* | Dipeptidyl-peptidase 4 | Up | 4.76 | 2.25 | 9.81 x 10^-6^ | 5.13 x 10^-6^ |
| NM_025061 | *LRRC8E* | Leucine rich repeat containing 8 family, member E | Up | 4.73 | 2.24 | 2.23 x 10^-5^ | 1.21 x 10^-5^ |
| NM_024337 | *IRX1* | Iroquois homeobox 1 | Up | 4.68 | 2.23 | 1.18 x 10^-5^ | 6.23 x 10^-6^ |
| NM_138455 | *CTHRC1* | Collagen triple helix repeat containing 1 | Up | 4.65 | 2.22 | 4.4 x 10^-5^ | 2.47 x 10^-5^ |
| AJ295982 |  |  | Up | 4.62 | 2.21 | 7.65 x 10^-6^ | 3.99 x 10^-6^ |
| NM_138461 | *TM4SF19* | Transmembrane 4 L six family member 19 | Up | 4.59 | 2.20 | 1.08 x 10^-4^ | 6.33 x 10^-5^ |
| NM_031913 | *ESYT3* | Extended synaptotagmin-like protein 3 | Up | 4.50 | 2.17 | 1.1 x 10^-4^ | 6.48 x 10^-5^ |
| NM_001145715 | *KPNA7* | Karyopherin alpha 7 (importin alpha 8) | Up | 4.48 | 2.16 | 8.21 x 10^-6^ | 4.32 x 10^-6^ |
| NM_025074 | *FRAS1* | Fraser syndrome 1 | Up | 4.46 | 2.16 | 1.31 x 10^-5^ | 7.06 x 10^-6^ |
| NM_001742 | *CALCR* | Calcitonin receptor | Up | 4.44 | 2.15 | 1.43 x 10^-4^ | 8.54 x 10^-5^ |
| AK096928 |  |  | Up | 4.42 | 2.15 | 6.46 x 10^-5^ | 3.73 x 10^-5^ |
| NM_001129742 | *CALHM3* | Calcium homeostasis modulator 3 | Up | 4.42 | 2.14 | 1.04 x 10^-4^ | 1.04 x 10^-4^ |
| NM_001146188 | *TOX3* | TOX high mobility group box family member 3 | Up | 4.36 | 2.12 | 1.24 x 10^-4^ | 1.24 x 10^-4^ |
| NM_004460 | *FAP* | Fibroblast activation protein, alpha | Up | 4.33 | 2.11 | 5.15 x 10^-5^ | 2.95 x 10^-5^ |
|  |  |  | Up | 4.24 | 2.08 | 4.49 x 10^-8^ | 2.12 x 10^-8^ |
| NM_004717 | *DGKI* | Diacylglycerol kinase, iota | Up | 4.20 | 2.07 | 2.38 x 10^-5^ | 1.32 x 10^-5^ |
| NM_002839 | *PTPRD* | Protein tyrosine phosphatase, receptor type, D | Up | 4.18 | 2.06 | 1.36 x 10^-4^ | 8.19 x 10^-5^ |
| NM_020962 | *IGDCC4* | Immunoglobulin superfamily, DCC subclass, member 4 | Up | 4.14 | 2.05 | 8.49 x 10^-5^ | 5.29 x 10^-5^ |
| NM_031302 | *GLT8D2* | Glycosyltransferase 8 domain containing 2 | Up | 4.10 | 2.04 | 7.25 x 10^-5^ | 4.26 x 10^-5^ |
| NM_001007237 | *IGSF3* | Immunoglobulin superfamily, member 3 | Up | 4.09 | 2.03 | 1.38 x 10^-5^ | 7.60 x 10^-6^ |
| NM_207645 | *C11orf87* | Chromosome 11 open reading frame 87 | Up | 4.09 | 2.03 | 4.09 x 10^-5^ | 2.34 x 10^-5^ |
| NM_194312 | *ESPNL* | Espin-like | Up | 3.99 | 2.00 | 9.85 x 10^-5^ | 5.89 x 10^-5^ |
| NM_001040708 | *HEY1* | Hes-related family bHLH transcription factor with YRPW motif 1 | Up | 3.97 | 1.99 | 2.92 x 10^-5^ | 1.66 x 10^-5^ |
| NM_030788 | *DCSTAMP* | Dendrocyte expressed seven transmembrane protein | Up | 3.92 | 1.97 | 2.79 x 10^-5^ | 1.59 x 10^-5^ |
| NM_014568 | *GALNT5* | Polypeptide N-acetylgalactosaminyltransferase 5 | Up | 3.91 | 1.97 | 6.38 x 10^-5^ | 3.76 x 10^-5^ |
| NR_110267 | *LOC101927619* | Uncharacterized LOC101927619 | Up | 3.89 | 1.96 | 4.22 x 10^-5^ | 2.45 x 10^-5^ |
| NM_015265 | *SATB2* | SATB homeobox 2 | Up | 3.84 | 1.94 | 2.65 x 10^-5^ | 1.52 x 10^-5^ |
| NM_000165 | *GJA1* | Gap junction protein, alpha 1, 43kDa | Up | 3.81 | 1.93 | 1.98 x 10^-7^ | 1.01 x 10^-7^ |
| NM_178833 | *SLC9B2* | Solute carrier family 9, subfamily B, member 2 | Up | 3.81 | 1.93 | 6.92 x 10^-5^ | 4.11 x 10^-5^ |
| NM_052909 | *PLEKHG4B* | Pleckstrin homology domain containing, family G | Up | 3.76 | 1.91 | 1.09 x 10^-4^ | 1.09 x 10^-4^ |
|  | *LINC00673* | Long intergenic non-protein coding RNA 673 | Up | 3.72 | 1.90 | 1.92 x 10^-6^ | 1.03 x 10^-6^ |
| NM_001123366 | *HMSD* | Histocompatibility (minor) serpin domain containing | Up | 3.67 | 1.88 | 1.11 x 10^-4^ | 1.11 x 10^-4^ |
| NM_003485 | *GPR68* | G protein-coupled receptor 68 | Up | 3.64 | 1.86 | 1.19 x 10^-4^ | 7.34 x 10^-5^ |
|  |  |  | Up | 3.53 | 1.82 | 9.90 x 10^-5^ | 9.90 x 10^-5^ |
| NM_001164737 | *CALCR* | Calcitonin receptor | Up | 3.51 | 1.81 | 6.34 x 10^-5^ | 3.83 x 10^-5^ |
| NM_003619 | *PRSS12* | Protease, serine, 12 (neurotrypsin, motopsin) | Up | 3.50 | 1.81 | 4.07 x 10^-5^ | 2.42 x 10^-5^ |
| NM_003247 | *THBS2* | Thrombospondin 2 | Up | 3.49 | 1.80 | 1.05 x 10^-4^ | 1.05 x 10^-4^ |
| NM_001258248 | *SP6* | Sp6 transcription factor | Up | 3.49 | 1.80 | 1.07 x 10^-6^ | 5.85 x 10^-4^ |
| NM_019012 | *PLEKHA5* | Pleckstrin homology domain containing, family A member 5 | Up | 3.40 | 1.76 | 1.48 x 10^-5^ | 8.66 x 10^-6^ |
|  |  |  | Up | 3.39 | 1.76 | 2.19 x 10^-5^ | 1.29 x 10^-5^ |
| NM_005715 | *UST* | Uronyl-2-sulfotransferase | Up | 3.35 | 1.75 | 6.69 x 10^-5^ | 4.10 x 10^-5^ |
| NM_152621 | *SGMS2* | Sphingomyelin synthase 2 | Up | 3.35 | 1.75 | 5.20 x 10^-5^ | 3.16 x 10^-5^ |
| NM_004994 | *MMP9* | Matrix metallopeptidase 9 | Up | 3.33 | 1.74 | 9.61 x 10^-5^ | 5.97 x 10^-5^ |
| NM_012223 | *MYO1B* | Myosin IB | Up | 3.33 | 1.74 | 2.24 x 10^-5^ | 1.33 x 10^-5^ |
| NM_181342 | *FKBP7* | FK506 binding protein 7 | Up | 3.29 | 1.72 | 1.05 x 10^-4^ | 1.05 x 10^-4^ |
| NM_001542 | *IGSF3* | Immunoglobulin superfamily, member 3 | Up | 3.26 | 1.71 | 1.45 x 10^-4^ | 1.45 x 10^-4^ |
| NM_016848 | *SHC3* | SHC (Src homology 2 domain containing) transforming protein 3 | Up | 3.24 | 1.70 | 3.12 x 10^-5^ | 1.89 x 10^-5^ |
|  | *XLOC_005452* |  | Up | 3.23 | 1.69 | 2.96 x 10^-5^ | 1.79 x 10^-5^ |
| NM_001124758 | *SPNS2* | Spinster homolog 2 (Drosophila) | Up | 3.22 | 1.69 | 4.51 x 10^-5^ | 2.76 x 10^-5^ |
| NR_034143 | *CASC14* | Cancer susceptibility candidate 14 (non-protein coding) | Up | 3.22 | 1.69 | 4.80 x 10^-6^ | 2.81 x 10^-6^ |
|  | *LINC00605* | Long intergenic non-protein coding RNA 605 | Up | 3.15 | 1.66 | 4.86 x 10^-5^ | 3.01 x 10^-5^ |
| NM_024312 | *GNPTAB* | N-acetylglucosamine-1-phosphate transferase, alpha and beta subunits | Up | 3.13 | 1.65 | 2.72 x 10^-5^ | 1.66 x 10^-5^ |
| NM_018076 | *ARMC4* | Armadillo repeat containing 4 | Up | 3.10 | 1.63 | 8.24 x 10^-6^ | 4.96 x 10^-6^ |
| NM_005940 | *MMP11* | Matrix metallopeptidase 11 (stromelysin 3) | Up | 3.08 | 1.62 | 1.07 x 10^-4^ | 1.07 x 10^-4^ |
| NM_014398 | *LAMP3* | Lysosomal-associated membrane protein 3 | Up | 3.07 | 1.62 | 1.44 x 10^-4^ | 1.44 x 10^-4^ |
| XM_006724937 | *LOC100132705* | Immunoglobulin superfamily member 3-like | Up | 3.06 | 1.61 | 8.57 x 10^-5^ | 5.44 x 10^-5^ |
| NM_019854 | *PRMT8* | Protein arginine methyltransferase 8 | Up | 3.00 | 1.58 | 6.46 x 10^-5^ | 4.10 x 10^-5^ |
| TCONS_00030032 |  |  | Up | 2.96 | 1.57 | 2.28 x 10^-5^ | 1.43 x 10^-5^ |
| NM_006307 | *SRPX* | Sushi-repeat containing protein, X-linked | Up | 2.94 | 1.55 | 1.15 x 10^-5^ | 7.18 x 10^-6^ |
| NM_003034 | *ST8SIA1* | ST8 alpha-N-acetyl-neuraminide alpha-2,8-sialyltransferase 1 | Up | 2.89 | 1.53 | 1.45 x 10^-4^ | 9.54 x 10^-5^ |
| NM_017671 | *FERMT1* | Fermitin family member 1 | Up | 2.89 | 1.53 | 7.22 x 10^-5^ | 4.67 x 10^-5^ |
| NM_001081 | *CUBN* | Cubilin (intrinsic factor-cobalamin receptor) | Up | 2.88 | 1.53 | 1.24 x 10^-5^ | 7.78 x 10^-6^ |
| NM_030794 | *TDRD3* | Tudor domain containing 3 | Up | 2.85 | 1.51 | 6.78 x 10^-6^ | 4.28 x 10^-6^ |
| XR_108587 |  |  | Up | 2.79 | 1.48 | 2.26 x 10^-5^ | 1.46 x 10^-5^ |
| NM_000943 | *PPIC* | Peptidylprolyl isomerase C (cyclophilin C) | Up | 2.79 | 1.48 | 5.40 x 10^-6^ | 3.47 x 10^-6^ |
| NM_005264 | *GFRA1* | GDNF family receptor alpha 1 | Up | 2.75 | 1.46 | 8.36 x 10^-5^ | 5.53 x 10^-5^ |
| NM_016184 | *CLEC4A* | C-type lectin domain family 4, member A | Up | 2.73 | 1.45 | 3.33 x 10^-5^ | 2.18 x 10^-5^ |
| NM_001024630 | *RUNX2* | Runt-related transcription factor 2 | Up | 2.72 | 1.44 | 1.55 x 10^-5^ | 1.01 x 10^-5^ |
| NM_030801 | *MAGED4B* | Melanoma antigen family D, 4B | Up | 2.70 | 1.43 | 3.50 x 10^-5^ | 2.31 x 10^-5^ |
| NM_001047 | *SRD5A1* | Steroid-5-alpha-reductase, alpha polypeptide 1 | Up | 2.63 | 1.40 | 1.20 x 10^-5^ | 8.05 x 10^-6^ |
| NM_003371 | *VAV2* | Vav 2 guanine nucleotide exchange factor | Up | 2.61 | 1.38 | 4.92 x 10^-5^ | 3.32 x 10^-5^ |
|  |  |  | Up | 2.61 | 1.38 | 1.12 x 10^-4^ | 7.63 x 10^-5^ |
| NM_033120 | *NKD2* | Naked cuticle homolog 2 (Drosophila) | Up | 2.58 | 1.37 | 1.23 x 10^-4^ | 1.23 x 10^-4^ |
| NM_001037582 | *SCD5* | Stearoyl-CoA desaturase 5 | Up | 2.58 | 1.37 | 1.39 x 10^-4^ | 9.58 x 10^-5^ |
| NM_002773 | *PRSS8* | Protease, serine, 8 | Up | 2.56 | 1.36 | 1.24 x 10^-4^ | 8.54 x 10^-5^ |
|  |  |  | Up | 2.56 | 1.36 | 1.06 x 10^-4^ | 1.06 x 10^-4^ |
| NM_001042481 | *FRMD6* | FERM domain containing 6 | Up | 2.46 | 1.30 | 7.90 x 10^-6^ | 5.66 x 10^-6^ |
| NM_181078 | *IL21R* | Interleukin 21 receptor | Up | 2.40 | 1.26 | 3.05 x 10^-5^ | 2.19 x 10^-5^ |
|  |  |  | Up | 2.34 | 1.23 | 2.75 x 10^-5^ | 2.02 x 10^-5^ |
|  |  |  | Up | 2.30 | 1.20 | 1.37 x 10^-6^ | 1.15 x 10^-6^ |
| NR_104131 | *LINC01057* | Long intergenic non-protein coding RNA 1057 | Up | 2.28 | 1.19 | 7.16 x 10^-6^ | 5.64 x 10^-6^ |
| NM_004881 | *TP53I3* | Tumor protein p53 inducible protein 3 | Up | 2.26 | 1.18 | 6.27 x 10^-5^ | 4.71 x 10^-5^ |
| NM_001024736 | *CD276* | CD276 molecule | Up | 2.20 | 1.14 | 1.06 x 10^-4^ | 8.11 x 10^-5^ |
| NM_183376 | *ARRDC4* | Arrestin domain containing 4 | Up | 2.18 | 1.12 | 8.55 x 10^-5^ | 6.60 x 10^-5^ |
| NM_006670 | *TPBG* | Trophoblast glycoprotein | Up | 2.17 | 1.12 | 6.44 x 10^-6^ | 5.49 x 10^-6^ |
| NM_001031695 | *RBFOX2* | RNA binding protein, fox-1 homolog (C. elegans) 2 | Up | 2.09 | 1.06 | 1.66 x 10^-5^ | 1.43 x 10^-5^ |
| NR_034110 | *TRAF3IP2-AS1* | TRAF3IP2 antisense RNA 1 | Up | 2.07 | 1.05 | 1.34 x 10^-4^ | 1.34 x 10^-4^ |
|  | *ZNF815P* | Zinc finger protein 815, pseudogene | Up | 1.77 | 0.82 | 1.42 x 10^-4^ | 1.44 x 10^-4^ |
| NM_000367 | *TPMT* | Thiopurine S-methyltransferase | Up | 1.75 | 0.81 | 1.09 x 10^-4^ | 1.15 x 10^-4^ |
| AK091525 |  |  | Up | 1.74 | 0.80 | 4.75 x 10^-5^ | 5.53 x 10^-5^ |
| NM_005765 | *ATP6AP2* | ATPase, H+ transporting, lysosomal accessory protein 2 | Up | 1.64 | 0.71 | 4.90 x 10^-5^ | 6.91 x 10^-5^ |
| NM_001605 | *AARS* | Alanyl-tRNA synthetase | Down | -1.59 | -0.67 | 5.46 x 10^-5^ | 8.47 x 10^-5^ |
| NM_198679 | *RAPGEF1* | Rap guanine nucleotide exchange factor (GEF) 1 | Down | 1.60 | -0.68 | 1.19 x 10^-4^ | 1.19 x 10^-4^ |
| NM_018093 | *WDR74* | WD repeat domain 74 | Down | -1.63 | -0.71 | 3.77 x 10^-5^ | 5.57 x 10^-5^ |
| NM_001039619 | *PRMT5* | Protein arginine methyltransferase 5 | Down | -1.64 | -0.72 | 3.30 x 10^-5^ | 4.86 x 10^-5^ |
| NM_016457 | *PRKD2* | Protein kinase D2 | Down | -1.66 | -0.73 | 3.11 x 10^-5^ | 4.46 x 10^-5^ |
| NM_006990 | *WASF2* | WAS protein family, member 2 | Down | -1.71 | -0.77 | 1.06 x 10^-4^ | 1.19 x 10^-4^ |
| XR_425288 | *LOC102725378* | Uncharacterized LOC102725378 | Down | -1.72 | -0.78 | 1.33 x 10^-4^ | 1.44 x 10^-4^ |
| NM_030927 | *TSPAN14* | Tetraspanin 14 | Down | -1.73 | -0.79 | 1.27 x 10^-4^ | 1.35 x 10^-4^ |
| NM_003768 | *PEA15* | Phosphoprotein enriched in astrocytes 15 | Down | -1.83 | -0.87 | 1.45 x 10^-4^ | 1.45 x 10^-4^ |
| NM_006058 | *TNIP1* | TNFAIP3 interacting protein 1 | Down | -1.83 | -0.87 | 1.45 x 10^-5^ | 1.66 x 10^-5^ |
| NM_002405 | *MFNG* | MFNG O-fucosylpeptide 3-beta-N-acetylglucosaminyltransferase | Down | -1.83 | -0.88 | 4.68 x 10^-5^ | 4.79 x 10^-5^ |
| NM_052862 | *RCSD1* | RCSD domain containing 1 | Down | -1.85 | -0.89 | 1.02 x 10^-4^ | 9.60 x 10^-5^ |
| NM_001114618 | *MGAT1* | Mannosyl (alpha-1,3-)-glycoprotein beta-1 | Down | -1.87 | -0.90 | 1.16 x 10^-4^ | 1.08 x 10^-4^ |
| NM_015680 | *CNPPD1* | Cyclin Pas1/PHO80 domain containing 1 | Down | -1.89 | -0.92 | 2.63 x 10^-5^ | 2.64 x 10^-5^ |
| NM_006247 | *PPP5C* | Protein phosphatase 5, catalytic subunit | Down | -1.90 | -0.92 | 1.35 x 10^-4^ | 1.35 x 10^-4^ |
| NM_000442 | *PECAM1* | Platelet/endothelial cell adhesion molecule 1 | Down | -1.92 | -0.94 | 1.42 x 10^-4^ | 1.25 x 10^-4^ |
| NM_014945 | *ABLIM3* | Actin binding LIM protein family, member 3 | Down | -1.96 | -0.97 | 1.96 x 10^-5^ | 1.87 x 10^-5^ |
| NM_001540 | *HSPB1* | Heat shock 27kDa protein 1 | Down | -2.00 | -1.00 | 6.86 x 10^-5^ | 5.90 x 10^-5^ |
| BC036832 |  |  | Down | -2.00 | -1.00 | 9.72 x 10^-5^ | 8.23 x 10^-5^ |
| NM_003768 | *PEA15* | Phosphoprotein enriched in astrocytes 15 | Down | -2.03 | -1.02 | 5.46 x 10^-5^ | 4.66 x 10^-5^ |
| NM_138352 | *SAMD1* | Sterile alpha motif domain containing 1 | Down | -2.09 | -1.07 | 1.19 x 10^-4^ | 9.47 x 10^-5^ |
| NM_014220 | *TM4SF1* | Transmembrane 4 L six family member 1 | Down | -2.14 | -1.10 | 3.01 x 10^-5^ | 2.44 x 10^-5^ |
| NM_001166208 | *SYNPO* | Synaptopodin | Down | -2.23 | -1.16 | 6.94 x 10^-5^ | 5.27 x 10^-5^ |
| NM_001243756 | *PXN* | Paxillin | Down | -2.29 | -1.19 | 2.39 x 10^-5^ | 1.81 x 10^-5^ |
| NM_004415 | *DSP* | Desmoplakin | Down | -2.29 | -1.19 | 1.51 x 10^-5^ | 1.16 x 10^-5^ |
| NM_002020 | *FLT4* | Fms-related tyrosine kinase 4 | Down | -2.30 | -1.20 | 9.34 x 10^-5^ | 6.87 x 10^-5^ |
| NM_152406 | *AFAP1L1* | Actin filament associated protein 1-like 1 | Down | -2.30 | -1.20 | 5.35 x 10^-6^ | 4.21 x 10^-6^ |
| NM_207627 | *ABCG1* | ATP-binding cassette, sub-family G (WHITE), member 1 | Down | -2.32 | -1.21 | 3.92 x 10^-6^ | 3.10 x 10^-6^ |
| NM_022153 | *C10orf54* | Chromosome 10 open reading frame 54 | Down | -2.34 | -1.22 | 1.18 x 10^-5^ | 8.85 x 10^-6^ |
| NM_080759 | *DACH1* | Dachshund family transcription factor 1 | Down | -2.34 | -1.23 | 7.57 x 10^-5^ | 5.51 x 10^-5^ |
| NM_007121 | *NR1H2* | Nuclear receptor subfamily 1, group H, member 2 | Down | -2.36 | -1.24 | 7.64 x 10^-5^ | 5.53 x 10^-5^ |
| NM_030927 | *TSPAN14* | Tetraspanin 14 | Down | -2.37 | -1.25 | 6.56 x 10^-5^ | 4.72 x 10^-5^ |
| NM_024726 | *IQCA1* | IQ motif containing with AAA domain 1 | Down | -2.41 | -1.27 | 8.43 x 10^-5^ | 6.01 x 10^-5^ |
| NM_001282714 | *FAM107A* | Family with sequence similarity 107, member A | Down | -2.42 | -1.27 | 1.19 x 10^-4^ | 1.19 x 10^-4^ |
| NM_003064 | *SLPI* | Secretory leukocyte peptidase inhibitor | Down | -2.49 | -1.31 | 1.54 x 10^-5^ | 1.08 x 10^-5^ |
|  | *XLOC_010730* |  | Down | -2.50 | -1.32 | 1.79 x 10^-5^ | 1.25 x 10^-5^ |
| NM_201566 | *SLC16A13* | Solute carrier family 16, member 13 | Down | -2.58 | -1.37 | 1.47 x 10^-4^ | 1.47 x 10^-4^ |
|  |  |  | Down | -2.61 | -1.38 | 1.32 x 10^-4^ | 9.03 x 10^-5^ |
| AF289611 | *LOC100128343* | Uncharacterized LOC100128343 | Down | -2.80 | -1.49 | 1.43 x 10^-4^ | 9.47 x 10^-5^ |
| NM_024726 | *IQCA1* | IQ motif containing with AAA domain 1 | Down | -2.92 | -1.54 | 1.27 x 10^-4^ | 8.29 x 10^-5^ |
| NM_024735 | *FBXO31* | F-box protein 31 | Down | -2.93 | -1.55 | 5.56 x 10^-5^ | 3.55 x 10^-5^ |
| NM_006613 | *GRAP* | GRB2-related adaptor protein | Down | -3.11 | -1.64 | 2.32 x 10^-5^ | 1.42 x 10^-5^ |
| NR_110914 | *TP53TG3C* | TP53 target 3C | Down | -3.38 | -1.76 | 2.96 x 10^-6^ | 1.67 x 10^-6^ |
| NM_006691 | *LYVE1* | Lymphatic vessel endothelial hyaluronan receptor 1 | Down | -3.61 | -1.85 | 1.01 x 10^-4^ | 6.16 x 10^-5^ |
| NR_027151 | *PRR26* | Proline rich 26 | Down | -3.71 | -1.89 | 9.64 x 10^-5^ | 9.64 x 10^-5^ |
| NM_004519 | *KCNQ3* | Potassium voltage-gated channel, KQT-like subfamily, member 3 | Down | -3.80 | -1.93 | 2.24 x 10^-5^ | 1.28 x 10^-5^ |
|  | *XLOC_001496* |  | Down | -3.99 | -2.00 | 1.45 x 10^-4^ | 1.45 x 10^-4^ |
| NM_001066 | *TNFRSF1B* | Tumor necrosis factor receptor superfamily, member 1B | Down | -4.03 | -2.01 | 3.06 x 10^-5^ | 1.74 x 10^-5^ |
| NM_002193 | *INHBB* | Inhibin, beta B | Down | -4.12 | -2.04 | 1.08 x 10^-4^ | 1.08 x 10^-4^ |
|  | *ALU1* | Alu 1 Element | Down | -5.02 | -2.33 | 3.17 x 10^-7^ | 1.44 x 10^-7^ |
| NM_025260 | *C6orf25* | Chromosome 6 open reading frame 25 | Down | -5.82 | -2.54 | 5.35 x 10^-6^ | 2.62 x 10^-6^ |
| NM_080429 | *AQP10* | Aquaporin 10 | Down | -6.92 | -2.79 | 6.26 x 10^-7^ | 2.62 x 10^-6^ |
| NM_005306 | *FFAR2* | Free fatty acid receptor 2 | Down | -7.29 | -2.87 | 5.63 x 10^-5^ | 3.06 x 10^-5^ |
| AB305916 | *TRBV28* | T Cell Receptor Beta Variable 28 | Down | -7.50 | -2.91 | 3.35 x 10^-6^ | 1.55 x 10^-6^ |
| NM_000517 | *HBA2* | Hemoglobin, alpha 2 | Down | -7.64 | -2.93 | 7.61 x 10^-7^ | 3.25 x 10^-7^ |
|  | *XLOC_014512* |  | Down | -7.99 | -3.00 | 2.74 x 10^-7^ | 1.1 x 10^-7^ |
| NM_000517 | *HBA2* | Hemoglobin, alpha 2 | Down | -8.20 | -3.04 | 5.59 x 10^-7^ | 2.33 x 10^-7^ |
| NM_016509 | *CLEC1B* | C-type lectin domain family 1, member B | Down | -8.24 | -3.04 | 1.03 x 10^-4^ | 2.33 x 10^-7^ |
| NM_002620 | *PF4V1* | Platelet factor 4 variant 1 | Down | -9.31 | -3.22 | 2.34 x 10^-6^ | 1.04 x 10^-6^ |
| NM_022468 | *MMP25* | Matrix Metallopeptidase 25 | Down | -9.33 | -3.22 | 4.32 x 10^-5^ | 2.28 x 10^-5^ |
| NR_120522 | *LOC102724484* | Uncharacterized LOC102724484 | Down | -10.04 | -3.33 | 1.01 x 10^-4^ | 5.6 x 10^-5^ |
| NM_001136503 | *SMIM24* | Small integral membrane protein 24 | Down | -10.29 | -3.36 | 1.38 x 10^-5^ | 6.73 x 10^-6^ |
| NM_030773 | *TUBB1* | Tubulin, beta 1 class VI | Down | -12.37 | -3.63 | 5.86 x 10^-7^ | 2.34 x 10^-7^ |
|  | *HSJ1167H4* |  | Down | -13.17 | -3.72 | 3.71 x 10^-6^ | 1.65 x 10^-6^ |
| NR_001552 | *TTTY16* | Testis-specific transcript, Y-linked 16 (non-protein coding) | Down | -13.65 | -3.77 | 6.28 x 10^-5^ | 3.34 x 10^-5^ |
| NR_047499 | *LINC00570* | Long intergenic non-protein coding RNA 570 | Down | -14.00 | -3.81 | 1.03 x 10^-4^ | 8.67 x 10^-5^ |
| NM_144673 | *CMTM2* | CKLF-like MARVEL transmembrane domain containing 2 | Down | -14.25 | -3.83 | 2.71 x 10^-5^ | 1.36 x 10^-5^ |
| NM_001557 | *CXCR2* | Chemokine (C-X-C motif) receptor 2 | Down | -14.93 | -3.90 | 9.27 x 10^-6^ | 4.34 x 10^-6^ |
| NM_000519 | *HBD* | Hemoglobin, delta | Down | -15.75 | -3.98 | 7.89 x 10^-8^ | 2.74 x 10^-8^ |
| NM_002100 | *GYPB* | Glycophorin B (MNS blood group) | Down | -16.15 | -4.01 | 1.03 x 10^-4^ | 1.43 x 10^-4^ |
| XM_005261527 | *SEC14L3* | SEC14-like 3 (S. cerevisiae) | Down | -16.65 | -4.06 | 2.98 x 10^-5^ | 1.5 x 10^-5^ |
| AK128128 | *FLJ46249* |  | Down | -16.90 | -4.08 | 6.19 x 10^-5^ | 3.27 x 10^-5^ |
| NM_016509 | *CLEC1B* | C-type lectin domain family 1, member B | Down | -17.06 | -4.09 | 1.34 x 10^-5^ | 6.39 x 10^-6^ |
| NM_016509 | *CLEC1B* | C-type lectin domain family 1, member B | Down | -17.67 | -4.14 | 4.83 x 10^-6^ | 2.15 x 10^-6^ |
| NM_002049 | *GATA1* | GATA binding protein 1 (globin transcription factor 1) | Down | -19.55 | -4.29 | 7.87 x 10^-5^ | 4.21 x 10^-5^ |
| NM_005764 | *PDZK1IP1* | PDZK1 interacting protein 1 | Down | -20.36 | -4.35 | 7.59 x 10^-6^ | 3.47 x 10^-6^ |
| NM_006163 | *NFE2* | Nuclear factor, erythroid 2 | Down | -22.54 | -4.49 | 3.22 x 10^-5^ | 1.62 x 10^-5^ |
|  | *XLOC_013489* |  | Down | -23.69 | -4.57 | 2.85 x 10^-5^ | 1.42 x 10^-5^ |
| NM_002619 | *PF4* | Platelet factor 4 | Down | -31.42 | -4.97 | 1.26 x 10^-7^ | 4.32 x 10^-8^ |
|  | *XLOC_000346* |  | Down | -31.94 | -5.00 | 1.26 x 10^-7^ | 2.56 x 10^-5^ |
| NM_000032 | *ALAS2* | Aminolevulinate, delta-, synthase 2 | Down | -33.49 | -5.07 | 1.93 x 10^-5^ | 9.3 x 10^-6^ |
| NM_005980 | *S100P* | S100 calcium binding protein P | Down | -33.56 | -5.07 | 1.11 x 10^-4^ | 6.06 x 10^-5^ |
| NM_005331 | *HBQ1* | Hemoglobin, theta 1 | Down | -34.07 | -5.09 | 3.58 x 10^-6^ | 1.53 x 10^-6^ |
| NM_002704 | *PPBP* | Pro-platelet basic protein (chemokine (C-X-C motif) ligand 7) | Down | -39.94 | -5.32 | 4.11 x 10^-8^ | 1.3 x 10^-8^ |
| NM_000517 | *HBA2* | Hemoglobin, alpha 2 | Down | -41.07 | -5.36 | 2.47 x 10^-7^ | 8.77 x 10^-8^ |
| NM_001003938 | *HBM* | Hemoglobin, mu | Down | -45.11 | -5.50 | 7.66 x 10^-5^ | 4.05 x 10^-5^ |
| NM_018437 | *HEMGN* | Hemogen | Down | -53.12 | -5.73 | 1.89 x 10^-6^ | 7.66 x 10^-7^ |
| NM_005621 | *S100A12* | S100 calcium binding protein A12 | Down | -56.95 | -5.83 | 7.25 x 10^-5^ | 3.81 x 10^-5^ |
| NM_005621 | *S100A12* | S100 calcium binding protein A12 | Down | -58.82 | -5.88 | 4.6 x 10^-5^ | 2.34 x 10^-5^ |
| NM_000559 | *HBG1* | Hemoglobin, gamma A | Down | -88.82 | -6.47 | 1.94 x 10^-6^ | 7.82 x 10^-7^ |
| Accession #: Accession Number. Symbol: Entity Symbol. ↑↓: Regulation. Abs FC: Absolute Fold Change. Log FC: Log transformed Fold Change. P Value^a^: Adjusted Student T-test P value for microarray corrected for multiple testing by the Bonferroni FWER method. P Value^b^: Adjusted Moderated T-test P value for microarray corrected for multiple testing by the Bonferroni FWER method. | | | | | | | |
